# Prestressed cells are prone to cytoskeleton failures under localized shear strain: an experimental demonstration on muscle precursor cells

**DOI:** 10.1038/s41598-018-26797-4

**Published:** 2018-06-05

**Authors:** Laura Streppa, Francesca Ratti, Evelyne Goillot, Anne Devin, Laurent Schaeffer, Alain Arneodo, Françoise Argoul

**Affiliations:** 1 Ecole Normale Supérieure de Lyon, CNRS, Laboratoire de Physique, UMR5672, F-69007 Lyon, France; 2 Université de Lyon 1, F-69100 Villeurbanne, France; 3 Ecole Normale Supérieure de Lyon, CNRS, LBMC, UMR5239, F-69007 Lyon, France; 4 Université de Bordeaux, CNRS, IBGC, UMR5095, F-33077 Bordeaux, France; 5 Université de Bordeaux, CNRS, LOMA, UMR5798, F-33405 Talence, France

## Abstract

We report on a wavelet based space-scale decomposition method for analyzing the response of living muscle precursor cells (C2C12 myoblasts and myotubes) upon sharp indentation with an AFM cantilever and quantifying their aptitude to sustain such a local shear strain. Beyond global mechanical parameters which are currently used as markers of cell contractility, we emphasize the necessity of characterizing more closely the local fluctuations of the shear relaxation modulus as they carry important clues about the mechanisms of cytoskeleton strain release. Rupture events encountered during fixed velocity shear strain are interpreted as local disruptions of the actin cytoskeleton structures, the strongest (brittle) ones being produced by the tighter and stiffer stress fibers or actin agglomerates. These local strain induced failures are important characteristics of the resilience of these cells, and their aptitude to maintain their shape via a quick recovery from local strains. This study focuses on the perinuclear region because it can be considered as a master mechanical organizing center of these muscle precursor cells. Using this wavelet-based method, we combine the global and local approaches for a comparative analysis of the mechanical parameters of normal myoblasts, myotubes and myoblasts treated with actomyosin cytoskeleton disruptive agents (ATP depletion, blebbistatin).

## Introduction

Living cells are active mechanical machines which can withstand forces and deformations and can adapt quite rapidly to their mechanical environment. This malleability is mediated by three major cytoskeleton (CSK) filament networks, namely microtubules (MTs), actin filaments (F-actin), and intermediate filaments (IFs)^1,2^. Among these three filament networks, the actin filaments are involved in many mechanical processes such as cellular reshaping, locomotion, substrate adhesion, phagocytosis and plasma membrane compartmentalization^3^, they henceforth have been assigned the role of active CSK organizer. Actin polymerization and actomyosin dynamics produce the driving motile force of eukaryotic cells (lamellipodia, filopodia, micro-spikes)^4^, they are both driven by ATP. Actin dynamics is tightly regulated in time and space by a considerable number of actin binding proteins (ABPs). Genetic defects and abnormal expression of ABPs are often related to congenital and acquired human diseases confirming their critical role in actin CSK dynamical regulation^5,6^. MFs are tracks for their ATP-driven myosin molecular motors. Among myosins, non-muscle myosins II (NMM II) are the principal actin CSK regulatory proteins^7^; they have an important role in cell shaping and motility^8^. The actomyosin apparatus acts as a mechanical tensor in the mechanical coupling of the CSK to the extra cellular matrix (ECM) *via* focal adhesions (FAs), in mechanotransduction of external stresses to the nucleus^9^, and in exertion of resistance against forces^3^. In particular, ventral stress fibers have a key role in mechanosensing^10^ and can be classified in (i) peripheral stress fibers running along the edges of adherent cells, and (ii) perinuclear stress fibers drapped over the nucleus^11^. Perinuclear caps have a protective and mechanical confining role for the underlying nuclei. Given that the nuclear membranes and their adjacent lamina network are very sensitive to disruptions and deformations, perinuclear caps are the guardians of their mechanical stability, ensuring a correct chromatin organization and assisting the cell cycle timing and nuclear machineries involving DNA^12^. Soft perinuclear zones withstanding rather large deformations without CSK rupture confer to the cell a ductility upon deformation and assist its shape recovery. Conversely, highly tensed perinuclear zones propitious to localized failures (brittle) by disruption of cross-linked CSK domains, impede a complete shape recovery after deformation. To distinguish and quantify these two situations, we took, as cell models, muscle precursor cells, namely myoblasts (C2C12) and their differentiated form in myotubes, and we tested their proneness to ductile or brittle failures in normal and altered growth media.

**Figure 1:**
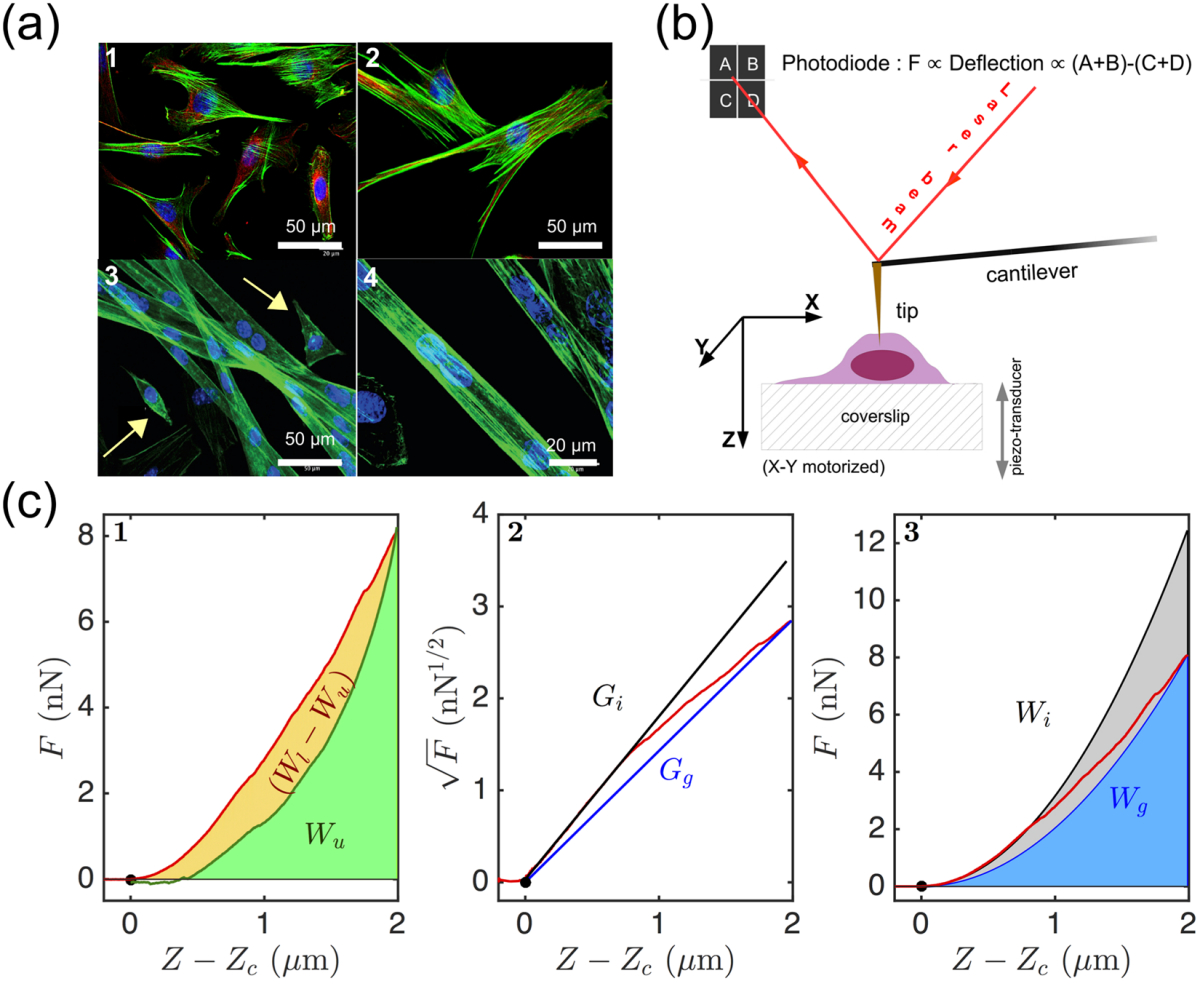
Principle of living cell indentation and FIC collection with an AFM cantilever tip. (**a**) Fluorescence images of myoblast cells (*Top*) and myotubes (*Bottom*) showing their nuclei (DAPI - blue), MFs (phalloidin alexa fluor 488 - green) and MTs (*β*-tubulin Cy3-conjugate - red) (see the Supplementary Information for further description of the staining). (**b**) Sketch of the AFM set-up. (**c**) Typical load (red) and unload (green) FICs collected on a myoblast with a 1 *μ*m/s cantilever scan velocity: (1) work integrals during load *W*_*l*_ and unload *W*_*u*_; (2) the square root of the FIC versus the distance to contact *Z* − *Z*_*c*_ highlights two linear regimes *G*_*g*_ and *G*_*i*_ bounding the loading FIC; (3) parabolic curves corresponding to *G*_*g*_ (resp. *G*_*i*_) and their work integrals *W*_*g*_ (resp. *W*_*i*_) (see the Section Methods).

C2C12 myoblast cells are immortalized cells derived from mouse satellite cells that can be switched to differentiation into myotubes by replacing their proliferation growth factor rich medium (GM) by a growth factor deprived medium (DM). After a few (~5) days in DM, confluent differentiated myoblasts fuse spontaneously and form syncitia of multinucleate myotubes^13^. C2C12 myoblasts can also be differentiated into adipocytes or osteoblasts when stimulated with suited nuclear transcription factors and other molecular cues^14,15^. When forced to adhesion on solid surfaces, myoblasts exhibit the characteristic spindle-shaped morphology, typical of mesenchymal cell lineage (Fig. 1(a) *Top*). Their morphology differs notably from the one of their differentiated myotubes which adopt elongated tubular shapes (Fig. 1(a) *Bottom*). Note that a small percentage of spindle-shaped unfused myoblasts can still be found among myotubes after 5 days of differentiation in DM. Adherent cells such as C2C12 myoblasts are mechanosensitive which means that the cell substrate for adhesion has a critical impact not only on their response to different stimuli, but also on their development, differentiation, disease, and regeneration^16^. While pulling on their sticky environment, these cells are capable to probe its elasticity and to a certain extent adapt their own elasticity to their environment^16^. C2C12 myoblasts can moreover adjust their myogenic differentiation to the substrate stiffness^17,18^. For the majority of tested cells requiring an anchoring on a surface, softer substrates lead to higher cell motility while stiffer substrates normally produce greater spreading and cell contractility^19,20^. Collagen gel coated surfaces are an exception in this context since, although classified as soft surfaces, they may still lead to maximal cell spreading^21^.

Single cell approaches have been widely developed in the past two decades to evaluate the mechanical properties of cells and their interplay with the extracellular environment, both in physiological and pathological conditions^22–24^. We chose a nano indentation technique (also called atomic force microscopy - AFM)^25–32^ to probe the mechanics of living myoblasts for its high spatial resolution and sensitivity. With this technique, the elastic (Young) modulus *E* of living cells was previously found to range from a few hundreds of Pa to hundreds of kPa. Varying the shape of the indentation probe tip can yield quite different Young modulus estimations^33^. Sharper tips (conical, pyramidal, single needle) produce a greater and more localized shearing and hence lead to higher Young’s modulus than spherical tips^33,34^. They are better suited to probe local (nanoscale) mechanical properties^35,36^ and to investigate local perturbations including disruptions of the CSK network. Spherical tips are instead used to estimate more global cell mechanical properties^33^. The Young’s modulus of muscle cells increases from myoblasts (the softest) to smooth, skeletal and cardiac muscles (the stiffest). This variability reflects also their strong adaptability to mechanical constraints and the variety of their *in vivo* organic functions. Actually, while myoblasts rather need high motility and deformability to migrate through the muscle tissue, cardiac or skeletal muscle cells instead require higher resistance against mechanical stress. Sharp (conical or pyramidal) indenters are better suited for the characterization of the spatial inhomogeneity of cell mechanics, and more precisely of their stress fiber resistance to deformation. This explains that we selected very sharp AFM tips (pyramidal shape) for the present study. Beyond a global characterization of the elasticity and viscosity of muscle precursor cells which can be estimated either from the work integrals of approach and retract force curves^37^ (as done here), or from cantilever position modulation experiments^38–40^, our study also focuses on local disruption events which have seldomly been evoked in such a context. Whereas the study of fracture mechanics and crack propagation in solid materials was developed during the beginning of the twentieth century^41^, these concepts were applied only recently to living organisms at large scales, *e*.*g*. bone fractures^42,43^, and there are still a few evidences of such phenomena in living cells^44^. A fracture occurs inside a strained material to release locally the strain, leading ultimately to a plastic irreversible deformation of the material (nonlinear regime). Upon deformation, an elastic material will store the elastic strain energy, up to the point of failure where this energy will be dissipated by plastic deformation or surface energy. In the case of living cells, this strain energy can also be released by viscous effects. As far as soft material mechanical failures are concerned, a lot of works have been performed on soft glassy materials or biopolymer gels. These failures originate from nonlinear elastoplastic or viscoplastic disassembly of cross-linked networks^45–47^. The global viscoplasticity of cells under large deformations has been studied by micron-scale stretching devices^48^. At these sub-micron scales, it appears that the destabilization of microtubules is more important and plays a major role in the viscoplasticity of cells. At nanometer-scales, and with a highly sensitive technique such as AFM, the local CSK actin filament network cohesion and its prestress determine the cell elasticity and its resilience to deformation^49^. The amplitude and strength of the local rupture events are not detectable by instrumental techniques performed at the level of the whole cell; this may explain why these behaviors were not much reported in previous works. In our experiments, we focused on the perinuclear region not only because it is the thickest part of an adherent cell but also because this area can be considered as a master mechanical organizing center. More precisely, we addressed the perinuclear actin cap firmness as a criteria for cell contractility and wellness of their actomyosin machinery. The muscle cell precursors were the best candidates to perform such a demonstration.

## Results

### Global mechanical characterization of Force Indentation Curves (FICs)

#### Nano-indentation of living cells with sharp tips

**Figure 2:**
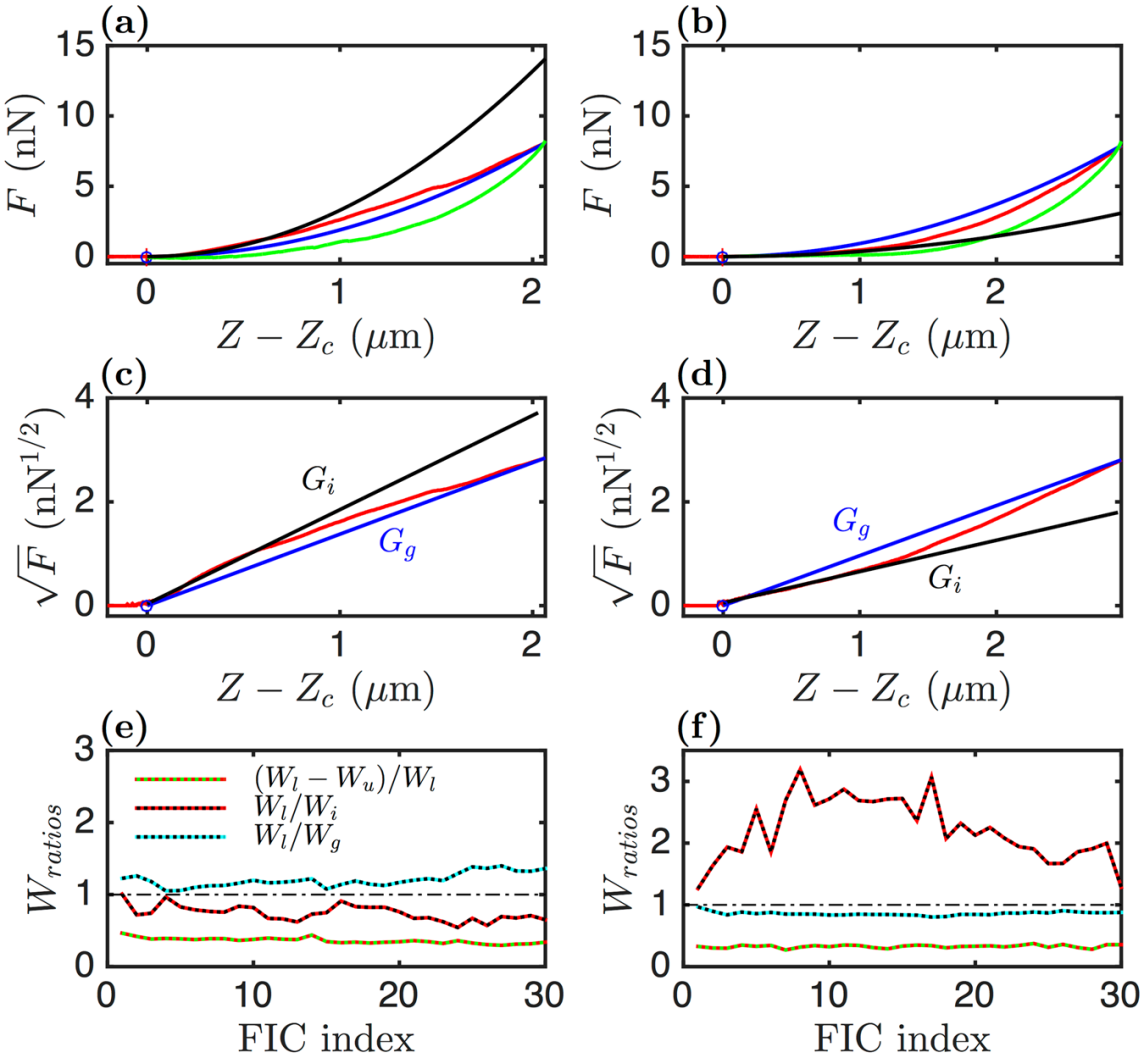
Global mechanical parameters extraction from FICs. FICs collected on two distinct (non interacting) myoblasts (left and right column) are shown. (**a**,**b**) Load (red) and unload (green) FICs and their parabolic fitting curves corresponding to global (*G*_*g*_, blue) and initial (*G*_*i*_, black) shear moduli. (**c**,**d**) Square root of the FICs and their linear fits with $\sqrt{\frac{4\,\tan \,\theta }{\pi (1-\nu )}{G}_{g}}(Z-{Z}_{c})$ and $\sqrt{\frac{4\,\tan \,\theta }{\pi (1-\nu )}{G}_{i}}(Z-{Z}_{c})$. (**e**,**f**) Temporal evolution of the work integral ratios *D*_*l*_ = (*W*_*l*_ − *W*_*u*_)/*W*_*l*_, *W*_*l*_/*W*_*i*_ and *W*_*l*_/*W*_*g*_ during 30 successive load-unload indentations on each cell.

AFM is a versatile technique which has been specifically used to collect the mechanical parameters of living adherent C2C12 myoblasts and myotubes^32,50^ (Fig. 1(a)). We used a constant velocity indentation mode in which the tip located at the far end of a micrometric cantilever (Fig. 1(b)) was moved toward the sample, along the vertical direction and at constant speed (*V*_0_) until a defined cantilever deflection value (*i*.*e*. a set-point force) was reached (position *Z*_*sp*_) (red loading curve in Fig. 1(c1)). Then the cantilever was withdrawn from the sample at the same constant speed (−*V*_0_) back to its starting Z position (green unloading curve in Fig. 1(c1)). These non-stationary fixed velocity indentation experiments allowed a rapid survey of the temporal changes of the living cell shear relaxation modulus *G*(*t*) which is related to the second derivative of the force indentation curves (FICs) (see the Section Methods and the Supplementary Information)^33,51–54^. More details on the cantilever calibration and the FICs can be found in the Supplementary Information. From the loading and unloading FICs, different parameters could be retrieved, such as the dissipation loss *D*_*l*_ (Eq. (6)), the initial shear modulus *G*_*i*_ (Eq. (7)), the global shear modulus *G*_*g*_ (Eq. (7)). Rapidly, we realized that for deformation depth greater than 500 nm, the amount of force that was necessary to perform a constant *V*_0_ cell indentation no longer followed a pure elastic response (Sneddon quadratic law^55^ (Eq. (5)). Indeed, the cells could behave either as a strain-softening (Figs 1(c2) and 2(c)) or as a strain-hardening (Fig. 2(d)) material. Plotting the square root of the force *F* versus the indentation *Z* − *Z*_*c*_ allows a fast discrimination of these situations (Fig. 2(c,d)). The two interpolated initial *G*_*i*_ and global *G*_*g*_ shear moduli circumvent the FIC. If *G*_*g*_ < *G*_*i*_ (or equivalently *W*_*g*_/*W*_*i*_ < 1) the cell behaves like a strain-softening material. Inversely, if *G*_*g*_ > *G*_*i*_ (or equivalently *W*_*g*_/*W*_*i*_ > 1) the cell behaves like a strain-hardening material.

By indenting the cells with sharp pyramidal tips, their extracellular membrane is also locally sheared and Ca^2+^ ion import into the cytoplasm can be promoted by either sharply localized pinches (Ca^2+^ ion diffusion through the membrane) or stretching and activation of nonselective cation channels (Ca^2+^ ion influx through the channels). This Ca^2+^ ion influx can occur both on the zone pinched by the cantilever tip or at some distance, corresponding to increased membrane tension. The typical time for a force-induced calcium influx was shown^56^ to be *t*_Ca_ ~ 2 s. Importantly, this accumulation of free Ca^2+^ ions was shown to precede a perinuclear actin assembly. When the initial stretch was not maintained, the increase of perinuclear actin indeed reached a maximum within about *t*_actin_ ~ 20–30 s. The remodeling of the actin cytoskeleton around the nucleus was therefore found^56^ to occur over time intervals longer than *t*_Ca_. This remodeling may involve specific actin binding proteins such as formins (INF2) that will reinforce mechanical protection of the nucleus by the perinuclear actin network^57^. If the reinforcement of the nuclear actin rim is triggered by Ca^2+^ ion influx, this process will likely lead to an apparent shear thickening of the force-indentation curves and to a possible amplification of the observed rupture events.

#### Global mechanical parameters of myoblasts and myotubes

**Figure 3:**
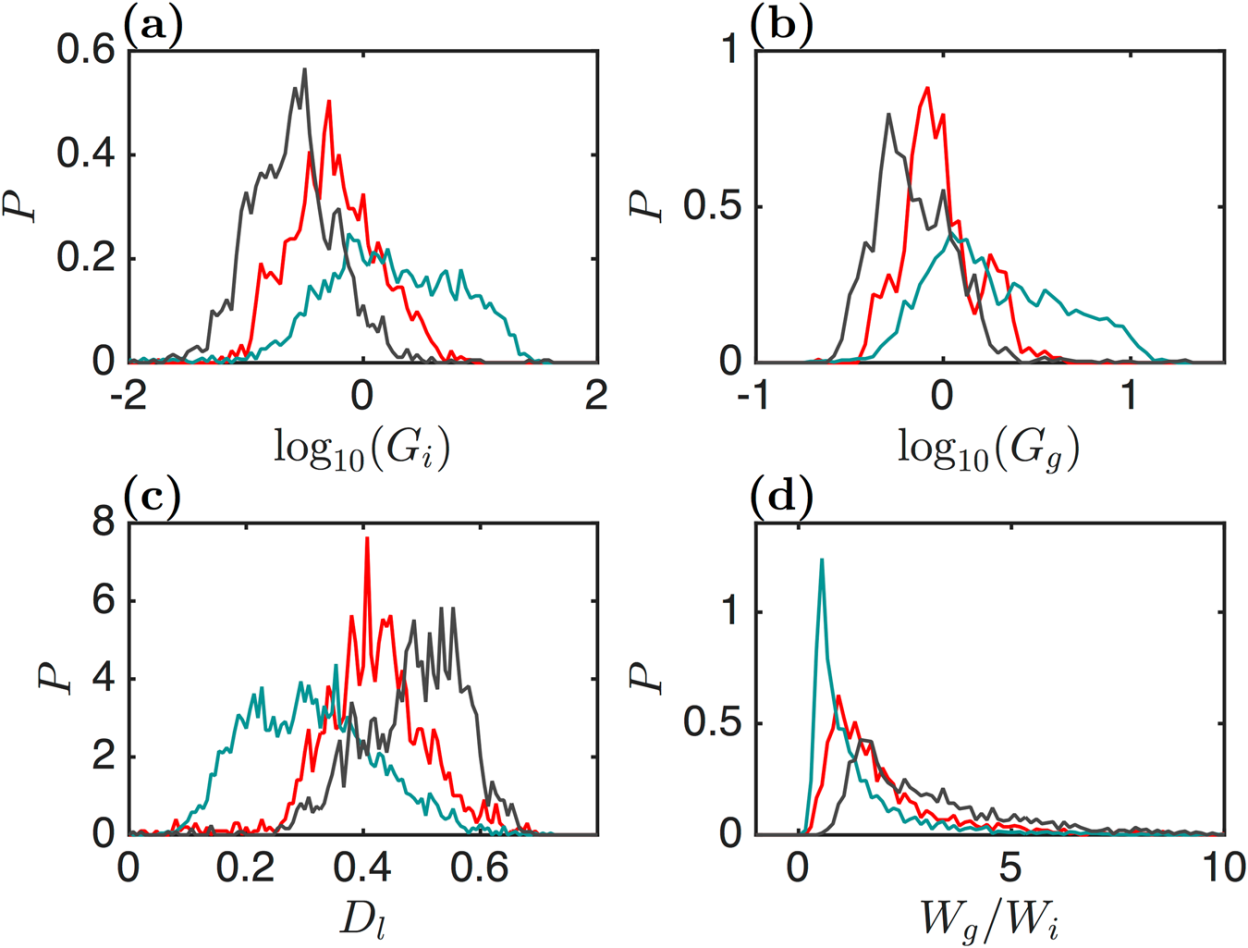
Distributions of the cell global mechanical parameters. These parameters were estimated for three sets of adherent cells: myoblasts (red, 54 cells), myotubes (blue, 56 cells) and ATP depleted myoblasts (black, 56 cells). (**a**) Initial elastic modulus *G*_*i*_ (kPa). (**b**) Global elastic modulus *G*_*g*_ (kPa). (**c**) Dissipative loss *D*_*l*_ upon load and unload indentations (Eq. (6)). (**d**) Ratio of interpolated elastic works *W*_*g*_ and *W*_*i*_ (Eq. (7)) (see the Section Methods).

Figure 3 gives the distributions of the four global mechanical parameters *G*_*i*_, *G*_*g*_, *D*_*l*_ and *W*_*g*_/*W*_*i*_ (See the Section Methods). The red plots were constructed from a set of 54 C2C12 myoblasts, with 30 FICs being recorded on each cell. The blue plots correspond to C2C12 myotubes, again with 30 FICs recorded on each cell. The two histograms of log_10_(*G*_*i*_) and log_10_(*G*_*g*_) (Fig. 3(a,b)) show that the myotubes are significantly stiffer than the myoblasts (Table 1): 〈*G*_*g*_〉 = 1.05 ± 0.08 kPa for the myoblasts as compared to 〈*G*_*g*_〉 = 2.02 ± 0.19 kPa for the myotubes, and also 〈*G*_*i*_〉 = 0.59 ± 0.07 kPa for the myoblasts as compared to 〈*G*_*i*_〉 = 1.66 ± 0.50 kPa for the myotubes. The mean of *D*_*l*_ values indicates that the indentation of myoblasts leads to a greater dissipative loss than the indentation of myotubes: 〈*D*_*l*_〉 = 0.42 ± 0.012 for the former and 〈*D*_*l*_〉 = 0.31 ± 0.015 for the latter. However, these mean values and their errors do not reflect the qualitative changes observed in the *D*_*l*_ value distribution from a nearly Gaussian distribution for the myoblasts to a much wider and asymmetric distribution for the myotubes (Fig. 3(c)). As far as their dissipative loss is concerned, the myotubes are therefore much more variable than the myoblasts. The distributions of *W*_*g*_/*W*_*i*_ values are clearly asymmetric in both cell types (Fig. 3(d)), with a very long exponential tail at larger values. The means and error bars are therefore not much informative. The position and intensity of their principal peak (*W*_*g*_/*W*_*i*_ = 0.55 for the myotubes and 0.94 for the myoblasts) and their medians are more representative of the cell mechanical deformability; the myotubes behave more closely to strain-softening materials (51% of the FICs have *W*_*g*_/*W*_*i*_ < 1), whereas the myoblasts behave more likely as strain-hardening materials (21.5% only of the FICs have *W*_*g*_/*W*_*i*_ < 1).

**Table 1:** Compilation of the mean values of the global parameters 〈*D*_*l*_〉, 〈*G*_*g*_〉, 〈*G*_*i*_〉 and 〈*W*_*g*_/*W*_*i*_〉 for C2C12 muscle precursor cells.

Type	N cells	N FICs	〈*D*_*l*_〉	〈*G*_*g*_〉 (kPa)	〈*E*_*g*_〉 (kPa)	〈*G*_*i*_〉 (kPa)	〈*W*_*g*_/*W*_*i*_〉
Myoblasts	54	1492	0.42 ± 0.01	1.05 ± 0.08	3.15 ± 0.24	0.59 ± 0.07	2.2 ± 0.3
Myotubes	56	1530	0.31 ± 0.02	2.02 ± 0.19	6.06 ± 0.57	1.66 ± 0.50	2.0 ± 0.7
Myoblasts ATP depleted	56	1248	0.48 ± 0.02	0.79 ± 0.10	2.37 ± 0.30	0.27 ± 0.06	3.7 ± 0.7
Myoblasts blebbistatin	23	534	0.27 ± 0.12	0.79 ± 0.10	2.37 ± 0.30	0.19 ± 0.06	3.2 ± 1.0

Four sets of cells are reported: normal C2C12 myoblasts, myotubes differentiated from C2C12 myoblasts, ATP depleted C2C12 myoblasts, and blebbistatin treated C2C12 myoblasts. The means are computed over all the FICS, and the uncertainties of these mean values correspond to the error of the mean, *i*.*e*. the ratio of the standard deviation by the square root of the number of cells, considering that each cell gives an independent measure. For comparison with other published works we also give the global Young modulus 〈*E*_*g*_〉 = 3〈*G*_*g*_〉 (assuming that the Poisson coefficient *ν* = 0.5). (See the Section Methods).

#### Alterations of the actomyosin network

We used two well-known methods to transform the actomyosin network, namely inhibition of ATP synthesis on the one hand and blebbistatin on the other hand. ATP deprivation is a well-known strategy that was often used to compare the impact of thermal (passive) and ATP-driven (active) fluctuations on living cell rheology^58–60^, the passive fluctuations being overwhelmed by the active fluctuations in the low frequency range (< a few tens of Hz)^61–63^. ATP deprived solutions (also called “rigour” solutions) were also shown to stiffen differentiated muscle fibers into a state similar to “rigour mortis” via the blockage of the muscle myosin II on actin filaments^64,65^. Without ATP, cross-linking of actin microfilaments by ADP-non muscle myosin II (ADP-NMMII) “freezes” the actomyosin network^66^. A complete ATP depletion (see the Section Methods for further details) can cause a 100-fold increase of the elastic modulus of the actomyosin network^66^. Interestingly, ATP depleted cells loose their typical intracellular actin organization but they retain their initial morphology (prior to depletion)^67–69^. Contradictory experiments also showed that ATP depleted cells could behave as a softer material^70,71^. Blebbistatin inhibits completely the NMMII motor activity by slowing down the phosphate release after ATP hydrolysis, thus setting NMMII in a weak actin-binding state and disassembling the stress fibers and the focal adhesions^72^. Fluorescence staining of the MT and MF corroborated these reported observations (Supplementary Figs S1 and S2).

The global mechanical parameter distributions for ATP depleted myoblasts are reported in Fig. 3 with black lines. Globally these cells behave much softer than both normal myoblasts and myotubes: 〈*G*_*g*_〉 = 0.79 ± 0.10 kPa (Fig. 3(b)) and 〈*G*_*i*_〉 = 0.27 ± 0.06 kPa (Fig. 3(a)). These cells are also liquid-like cells (〈*D*_*l*_〉 = 0.48 ± 0.02), and there is clearly a shift of the *D*_*l*_ histogram to larger values, with only a small percentage (<25%) of the FICs with a mean *D*_*l*_ value close to 0.4, typical of normal myoblasts, and a larger percentage (>50%) with *D*_*l*_ values above 0.5. Their ratio 〈*W*_*g*_/*W*_*i*_〉 is also very interesting since it increases dramatically to 3.7 ± 0.7, the fraction of FICs with *W*_*g*_/*W*_*i*_ < 1 dropping down to 2%. ATP depleted myoblasts are much softer, they dissipate more energy and surprisingly they behave as strain-hardening materials.

Blebbistatin has a similar impact on the global mechanical properties of myoblasts (Supplementary Fig. S7) 〈*G*_*g*_〉 = 0.79 ± 0.10 kPa and 〈*G*_*i*_〉 = 0.19 ± 0.06 kPa. But the distribution of dissipative loss *D*_*l*_ is quite different, since it decreases markedly to very low values (〈*D*_*l*_〉 = 0.27 ± 0.12), more than half of the FICs presenting *D*_*l*_ values around 0.2 (Supplementary Fig. S7(c)). Also the fraction of FICs with *W*_*g*_/*W*_*i*_ < 1 is very low (4%) (Supplementary Fig. S7(d)). These blebbistatin treated myoblasts are less dissipative on average and they can be classified within strain-hardening materials. Relying strictly on the global mechanical parameter estimations for myoblasts, myotubes and ATP depleted myoblasts, we could conclude that the stiffer cells are more prone to lower dissipative loss and strain-softening response.

**Figure 4:**
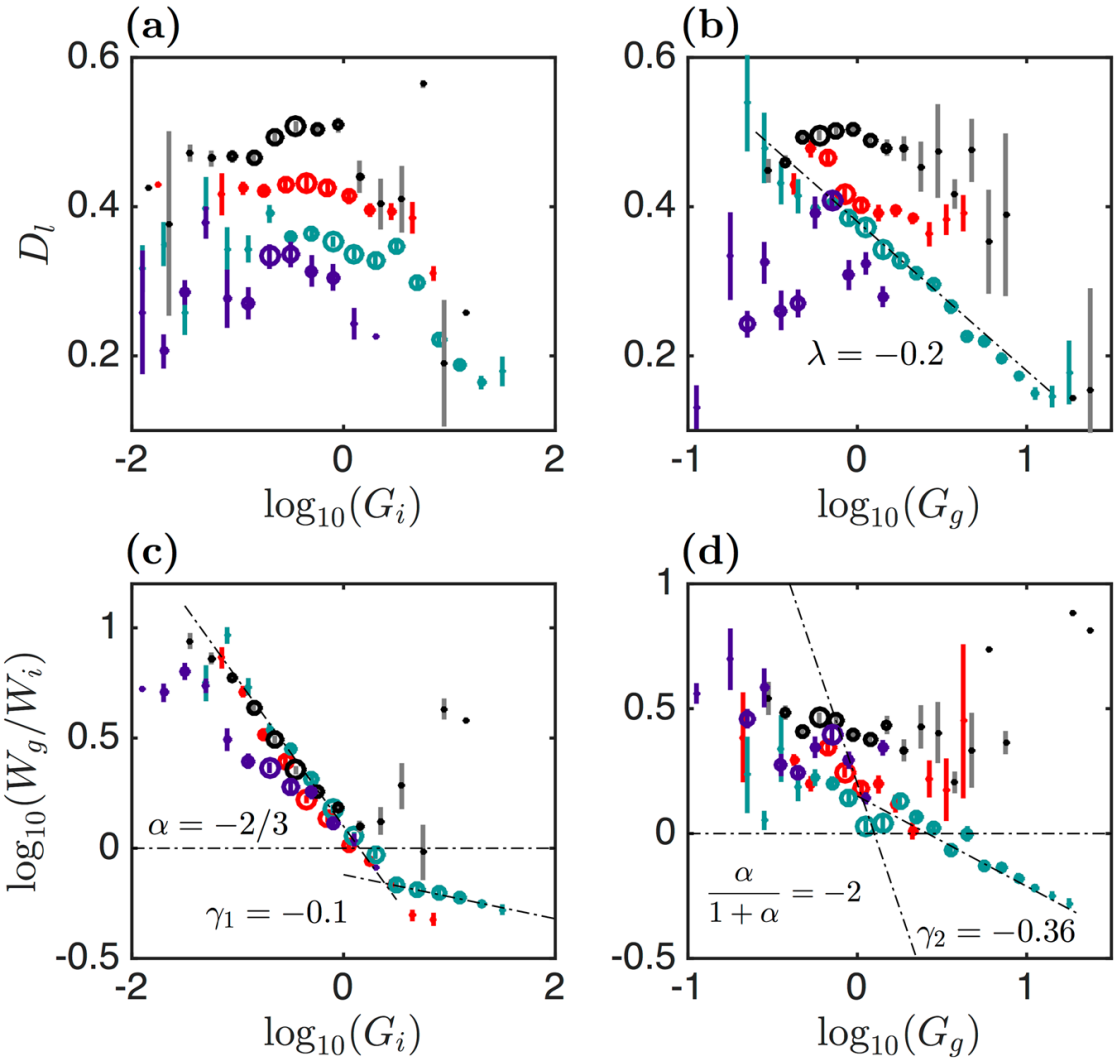
Cross-correlations of the global mechanical parameters. These global mechanical parameters were computed from the sets of myoblasts (red), myotubes (blue), ATP depleted (black) and blebbistatin treated (purple) myoblasts reported in Fig. 3 and Supplementrary S7. Box plots were reconstructed from fixed intervals of the two abscissa ${\mathrm{log}}_{10}({G}_{i})$ and ${\mathrm{log}}_{10}({G}_{g})$. (**a**) *D*_*l*_ vs ${\mathrm{log}}_{10}({G}_{i})$. (**b**) *D*_*l*_ vs ${\mathrm{log}}_{10}({G}_{g})$. (**c**) ${\mathrm{log}}_{10}({W}_{g}/{W}_{i})$ vs ${\mathrm{log}}_{10}({G}_{i})$. (**d**) ${\mathrm{log}}_{10}({W}_{g}/{W}_{i})$ vs ${\mathrm{log}}_{10}({G}_{g})$. The vertical lines give the error of the mean of each quantity. The circle diameters are proportional to the percent of FICs with a given *G*_*i*_ (**a**,**c**) or *G*_*g*_ (**b**,**d**). *G*_*i*_ and *G*_*g*_ are expressed in kPa units.

To elaborate on possible cross-correlations of the global mechanical parameters, we plotted (logarithmic representation) in Fig. 4 the values of *D*_*l*_ and *W*_*g*_/*W*_*i*_ versus *G*_*i*_ and *G*_*g*_ (in kPa) for the same set of cells as considered in Fig. 3. We cut the range of ${\mathrm{log}}_{10}({G}_{i})$ (resp. ${\mathrm{log}}_{10}({G}_{g})$) values in small intervals of 0.2 (resp. 0.1) and for each segment we reported the mean of *D*_*l*_ or *W*_*g*_/*W*_*i*_ with a colour dot, its error with a vertical line and for each interval we added a circle with a size proportional to the percent of FICs corresponding to this interval (in *G*_*i*_ or *G*_*g*_). Considering the most probable values of *D*_*l*_ and *W*_*g*_/*W*_*i*_ (highlighted by the largest circles), we observe that, for all cell types, *D*_*l*_ decreases very slightly with ${\mathrm{log}}_{10}\,{G}_{i}$ (Fig. 4(a)) (at least for *G*_*i*_ < 2 kPa), and that these plateaus match the *G*_*i*_ values of the histogram maxima in Fig. 3 and Supplementary Fig. S7. A similar conclusion could be made for the *D*_*l*_ vs ${\mathrm{log}}_{10}({G}_{g})$ representation in Fig. 4(b) for the myoblasts, whatever their culture medium (GM, ATP depleted or blebbistatin). Surprisingly, the circles of *D*_*l*_ vs ${\mathrm{log}}_{10}({G}_{g})$ for the myotubes align quite impressively on a straight line of slope *λ* ~ −0.2, meaning that these two quantities are strongly correlated for the myotubes (over more than a decade of *G*_*g*_ values). Indeed ${G}_{g}\sim {G}_{g}^{\ast }\,{10}^{-\frac{{D}_{l}}{|\lambda |}}$ decreases exponentially versus *D*_*l*_ with a characteristic decay factor |*λ*| = 0.2.

Another amazing alignment is found on the ${\mathrm{log}}_{10}({W}_{g}/{W}_{i})$ versus ${\mathrm{log}}_{10}\,{G}_{i}$ plots for *G*_*i*_ < 2 kPa (Fig. 4(c)), and this for both the myoblasts and myotubes, independently of their culture condition (except the blebbistatin treated myoblasts). This power law can be written as ${W}_{g}/{W}_{i}={G}_{g}/{G}_{i}\sim {G}_{i}^{\alpha }$ or ${G}_{g}\sim {G}_{i}^{1+\alpha }\sim {G}_{i}^{1/3}$ when considering the value *α* = −2/3 as suggested by the dashed straight line in Fig. 4(c). This scaling law is confirmed by a direct plot of ${\mathrm{log}}_{10}({G}_{g})$ vs ${\mathrm{log}}_{10}({G}_{i})$ (Supplementary Fig. S8). Plotting ${\mathrm{log}}_{10}({W}_{g}/{W}_{i})$ versus ${\mathrm{log}}_{10}({G}_{g})$ (Fig. 4(d)), we then expect that ${W}_{g}/{W}_{i}\sim {G}_{i}^{\alpha }\sim {G}_{g}^{\frac{\alpha }{1+\alpha }}={G}_{g}^{-2}$. Although less apparent, this scaling law can be observed when focusing on the most significant (large circles) *G*_*g*_ values. For the myotubes, we also notice that at larger values of *G*_*i*_, ${W}_{g}/{W}_{i}\sim {G}_{i}^{{\gamma }_{1}}\sim {G}_{i}^{-0.1}$ (Fig. 4(c)). This power law is again confirmed when plotting ${\mathrm{log}}_{10}({G}_{g})$ vs ${\mathrm{log}}_{10}({G}_{i})$ as ${G}_{g}\sim {G}_{i}^{1+{\gamma }_{1}}\sim {G}_{i}^{0.9}$ (Supplementary Fig. S8). With the same argument as before, we then expect an exponent ${\gamma }_{2}={\gamma }_{1}/(1+{\gamma }_{1})=-0.1/0.9\sim -\,0.11$ for *W*_*g*_/*W*_*i*_ versus *G*_*g*_, which deviates significantly from the exponent *γ*_2_ ~ −0.36 obtained at larger *G*_*g*_ values (Fig. 4(d)). This discrepancy actually results from the stiffest myotubes with a strain-softening-like response, located in the rightmost part of the histogram of *G*_*g*_ in Fig. 3(b) (blue line). As confirmed by the scatter plot of ${\mathrm{log}}_{10}({W}_{g}/{W}_{i})$ versus ${\mathrm{log}}_{10}({G}_{g})$ (Supplementary Fig. S9(a)), the distributions of *W*_*g*_/*W*_*i*_ (Fig. 3(d)) and ${\mathrm{log}}_{10}({G}_{g})$ (Fig. 3(b)) are far from symmetric, with fat tails at large values, which strongly bias the computation of the mean and standard deviation of these distributions. When computing the medians of these distributions, we obtain more consistent estimates of these exponents (*γ*_1_ ~ −0.17 and *γ*_2_ ~ −0.20).

Treating myoblasts with blebbistatin softened these cells and widened their global mechanical parameter histograms (Supplementary Fig. S7). The difficulty to perform mechanical indentation of the softest cells has somehow limited our sample size, and this questions the relevance of our estimated mean values which do not follow the power law behaviour of *W*_*g*_/*W*_*i*_ vs *G*_*i*_ for small values of *G*_*i*_ (Fig. 4(c)). Even if these cells were evaluated as softer and mimicking strain-hardening materials, they appear much less dissipative than expected (Supplementary Fig. S7(c)), as compared to the other myoblasts (GM and ATP depleted). This observation suggests that the higher dissipation loss of normal and ATP depleted myoblasts results from mechanisms different from pure viscous dissipation. Looking more closely on the local fluctuations of the loading FICs helped us unravel this contradiction.

### Unscrambling the local dynamics of FICs

**Figure 5:**
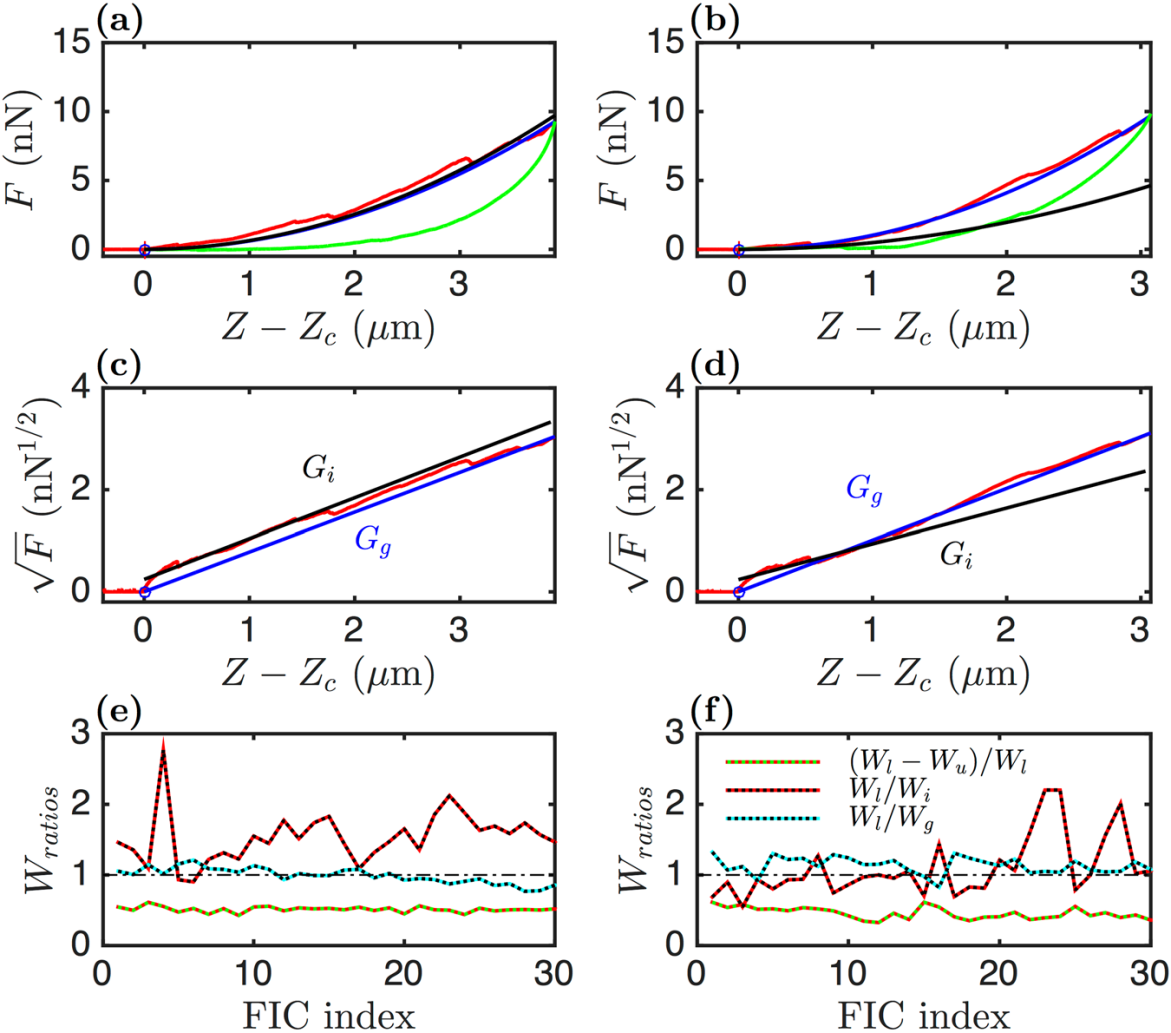
Local disruption events superimposed on global features of FICs. FICs collected on two distinct myoblasts (left and right column) are shown. (**a**,**b**) Load (red) and unload (green) FICs and their parabolic fitting curves corresponding to global (*G*_*g*_, blue) and initial (*G*_*i*_, black) shear moduli. (**c**,**d**) Square root of the FICs and their linear fits with $\sqrt{\frac{4\,\tan \,\theta }{\pi (1-\nu )}{G}_{g}}(Z-{Z}_{c})$ and $\sqrt{\frac{4\,\tan \,\theta }{\pi (1-\nu )}{G}_{i}}(Z-{Z}_{c})$. (**e**,**f**) Temporal evolution of the work integral ratios *D*_*l*_ = (*W*_*l*_ − *W*_*u*_)/*W*_*l*_, *W*_*l*_/*W*_*i*_ and *W*_*l*_/*W*_*g*_ during 30 successive load-unload indentations on each cell.

**Figure 6:**
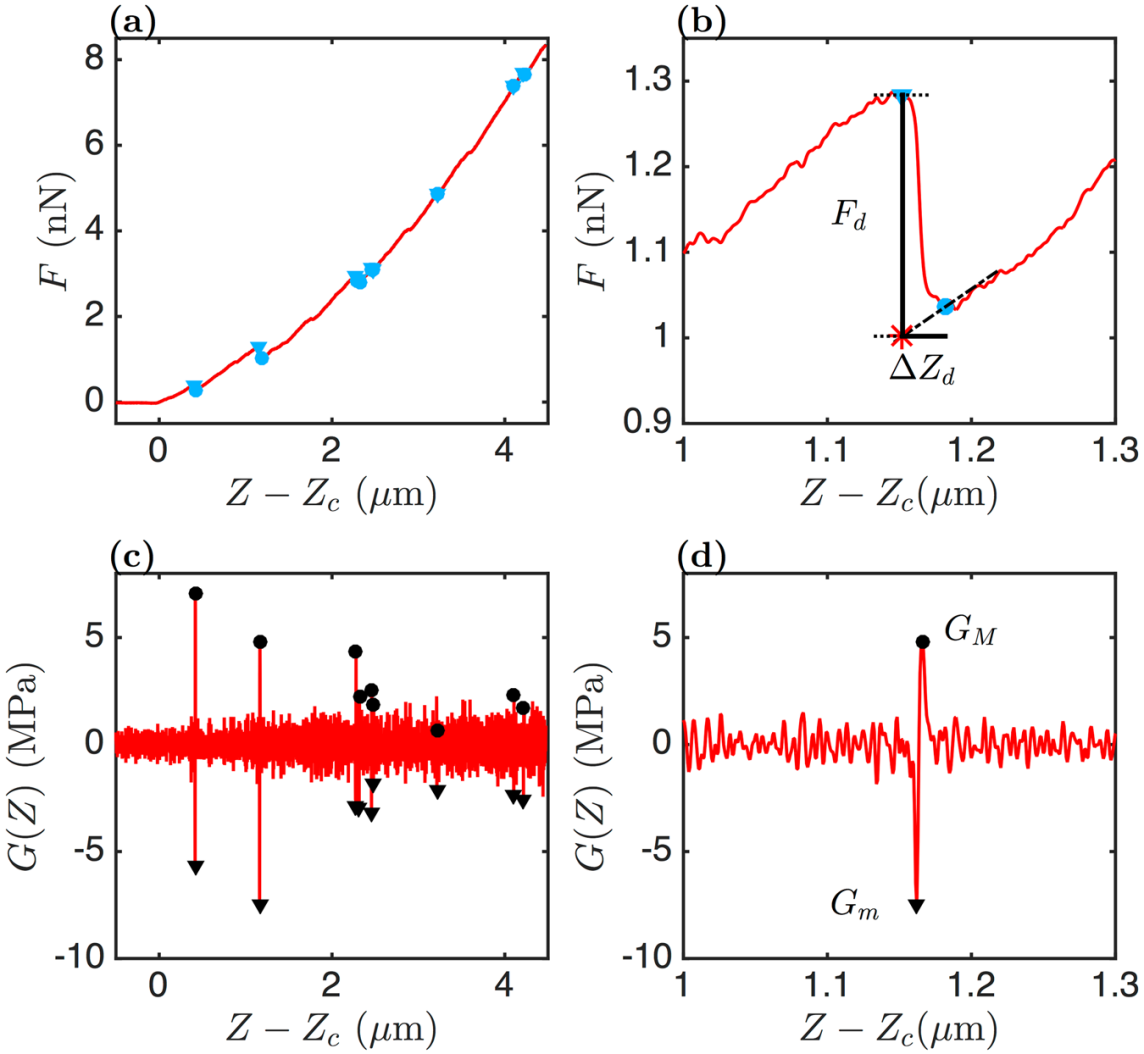
Detection of FIC disruption events. (**a**) Loading FIC collected on the perinuclear area of an adherent myoblast. (**b**) Zoom of (a) around a disruption event. (**c**) Second-order derivative *G*(*Z*) of the FIC (Eq. (3)) computed with a wavelet of size $w=2\sqrt{2}s=3.86$ nm (Eq. (4)). (**d**) Zoom of (**c**) around a disruption event. The minima *G*_*m*_ (resp. maxima *G*_*M*_) of *d*^2^*F*(*Z*)/*dZ*^2^ corresponding to a strong negative (resp. positive) curvature of the FIC are marked with black triangles (resp. dots). In a close neighbourhood of *G*_*m*_ and *G*_*M*_, the local maxima and minima of the FIC are detected and marked with blue triangles and dots respectively. The force drop *F*_*d*_ of a disruption event is corrected by taking into account the increase of the FIC (linear fit of the FIC, shown as a black dotted-dashed line in (**b**)).

The global characterization of FICs over length scales larger than several hundreds of nanometers provided a general overview of the mechanical properties of muscle precursor cells in different culture media. The power-law behaviour of *W*_*g*_/*W*_*l*_ vs *G*_*i*_ for all the myoblasts and myotubes (except for blebbistatin treated myoblasts) was very attractive because it suggested that we could build a general model for the mechanical response to strain of these cells. But we failed to extract a simple relation between the dissipation loss *D*_*l*_ and the global relaxation modulus *G*_*g*_ for myoblast cells, whereas we got a very nice exponential decay for the myotubes. This is an indication that we are missing some information that cannot be extracted from these large scale mechanical parameters. We therefore decided to focus on the local variations (fluctuations) of the FICs and we observed that in some situations (Fig. 5(a,b)), the FICs were showing local disruption events, with local risings of the force followed by sudden drops (Fig. 6(a,b)). We developed a wavelet-based space-scale detection method of these singular events that amounts to detect local curvature minima in the FICs (Fig. 6(c,d)) (Methods). For each disruption event, we computed the force drop *F*_*d*_, the penetration length corresponding to this drop Δ*Z*_*d*_ and the energy *E*_*d*_ = *F*_*d*_Δ*Z*_*d*_ (Fig. 6(b)). We also defined for each FIC a maximum drop energy $\widehat{{E}_{d}}$ corresponding to the mean energy of the three mostly energetic disruption events. When fewer (<3) disruption events were detected, we limited the mean to these events. FICs without disruption events were not included in the statistics.

#### Cross-correlations of disruption event energy with global mechanical parameters

**Figure 7:**
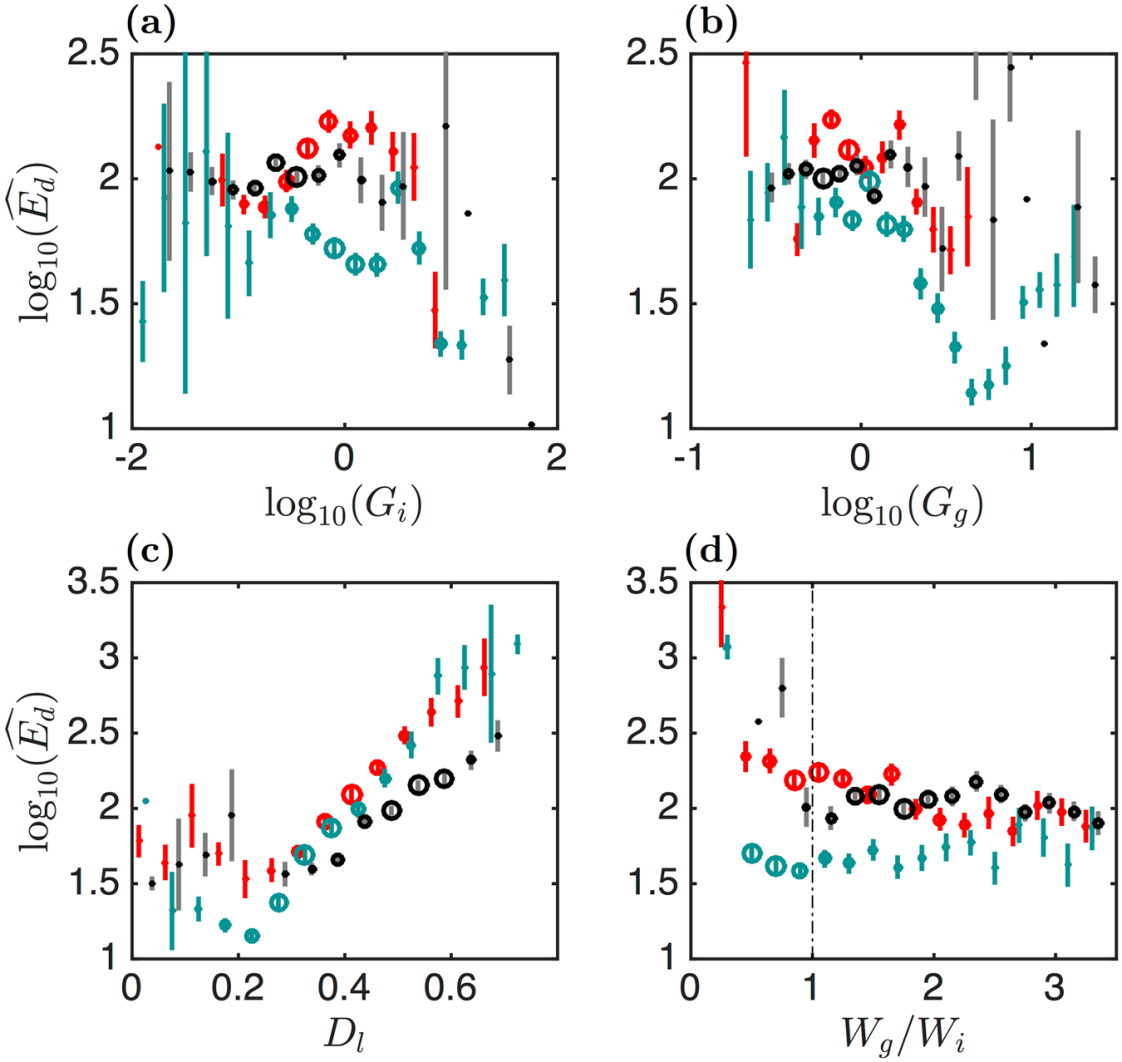
Cross-correlations of the local FIC disruption event maximal energies $\widehat{{E}_{d}}$ with global mechanical parameters. The same sets of cells (myoblasts, myotubes and ATP depleted myoblasts) were used as in Fig. 3. (**a**) ${\mathrm{log}}_{10}(\widehat{{E}_{d}})$ vs ${\mathrm{log}}_{10}({G}_{i})$. (**b**) ${\mathrm{log}}_{10}(\widehat{{E}_{d}})$ vs ${\mathrm{log}}_{10}({G}_{g})$. (**c**) ${\mathrm{log}}_{10}(\widehat{{E}_{d}})$ vs *D*_*l*_. (**d**) ${\mathrm{log}}_{10}(\widehat{{E}_{d}})$ vs *W*_*g*_/*W*_*i*_. Same representation and color coding as in Fig. 4. *G*_*i*_ and *G*_*g*_ are expressed in kPa, and $\widehat{{E}_{d}}$ in *k*_*B*_*T* units.

We first constructed cross-correlation plots of local disruption event energy with the global mechanical quantities *G*_*i*_, *G*_*g*_, *D*_*l*_ and *W*_*g*_/*W*_*i*_, discussed in the previous sections (Fig. 7). We did not include the plots for the blebbistatin treated myoblasts because the percentage of FICs with disruption events was too small (less than 4% of the FICs showed such events). As shown in Fig. 7(a,b), the disruption events reach much greater energies $\widehat{{E}_{d}}$ for myoblasts and ATP depleted myoblasts than myotubes, and these energy maxima occurred for values of *G*_*i*_ and *G*_*g*_ smaller than 1 kPa. These energy maxima values for myoblasts strikingly fall down for *G*_*i*_ and *G*_*g*_ $\gtrsim$ 2 kPa. For the myotubes, much smaller values of $\widehat{{E}_{d}}$ are found in the *G*_*i*_ and *G*_*g*_ middle range values. Indeed, smaller $\widehat{{E}_{d}}$ values are found in the middle range *G*_*g*_ values than in the corresponding *G*_*i*_ range. This probably means that sorting out the FICs with small intervals of *G*_*i*_ or *G*_*g*_ are not equivalent. We clearly delimitate two regimes in the cross-correlation plots of ${\mathrm{log}}_{10}({G}_{g})$ vs ${\mathrm{log}}_{10}({G}_{i})$ (Supplementary Fig. S8(b)), at the boundary of which the two regimes overlap and the estimation of $\widehat{{E}_{d}}$ may be misleading. Nevertheless, it is clear from Fig. 7(a,b,d) that the myotubes develop much weaker rupture events upon indentation than the myoblasts (in GM and ATP depletion buffer). Surprisingly we also notice that those of the myotubes with largest *G*_*i*_ and *G*_*g*_ values have also weaker (lowest energy) rupture events, as confirmed in the cross-correlation plot of ${\mathrm{log}}_{10}(\widehat{{E}_{d}})$ vs *D*_*l*_ (Fig. 7(c)). Given that these events of highest *G*_*i*_ and *G*_*g*_ values correspond to low dissipative loss *D*_*l*_ (Fig. 4(b)), we suspect that if the rupture events would be part of this dissipative loss, they would release less mechanical energy. These lower energy rupture events likely correspond to ductile failure events of the actin CSK during indentation. Conversely, higher energy events observed on FICs with much higher dissipative loss values (Fig. 7(c)) instead correspond to more abrupt and more energetic brittle failure events of the actin CSK network. The greater the energy of the rupture event, the higher the dissipative loss. The fact that the two ${\mathrm{log}}_{10}(\widehat{{E}_{d}})$ vs *D*_*l*_ plots for myotubes and normal myoblasts get closer to each other in the *D*_*l*_ interval [0.3; 0.6] seems to be in contradiction with the other plots of ${\mathrm{log}}_{10}(\widehat{{E}_{d}})$ vs ${\mathrm{log}}_{10}({G}_{g})$, ${\mathrm{log}}_{10}({G}_{i})$ and *W*_*g*_/*W*_*i*_ (Fig. 7). Actually, we think instead that as far as the rupture events are considered, the best quantity to distinguish ductile from brittle failure events is *D*_*l*_ and not the global mechanical parameters *G*_*i*_ and *G*_*g*_. Greater force disruption events reflect a very local stiffening of a small CSK domain penetrated by the cantilever tip, followed by a sudden force drop. These major events are rather due to a local inhomogeneity of the mechanical strength of the cell than to a global mechanical characteristics of the cell. The rupture events of highest energy correspond to strain-stiffening-like FICs (Fig. 7(d)). We anticipate that higher prestressed cells which develop thicker and tenser stress fibers will be very good candidates to develop higher energy rupture events.

#### Statistical analysis of the force drops and energies of disruption events

**Figure 8:**
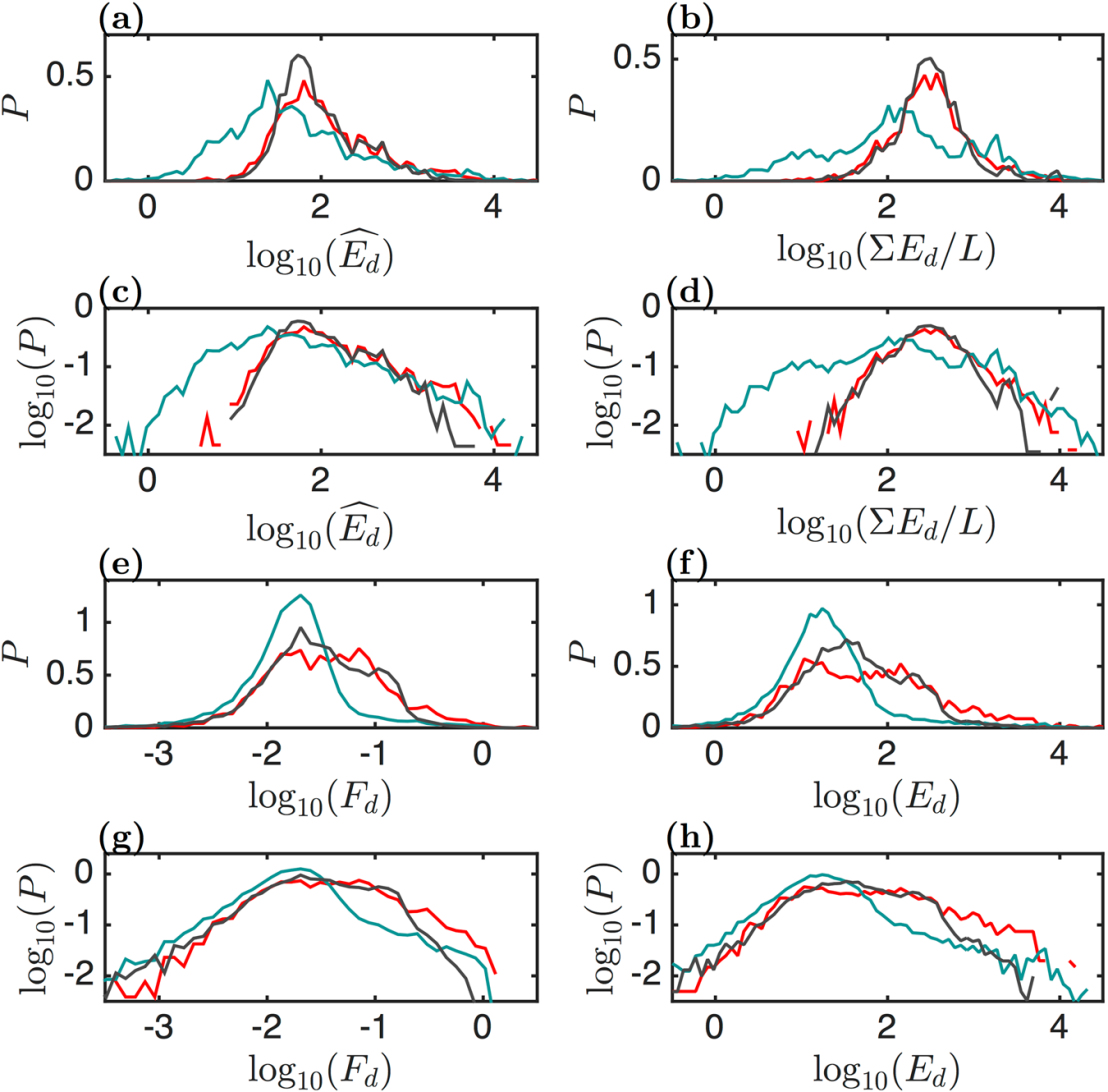
Distributions of the local force drop F_d_ (nN) and released energy E_d_ (k_B_T) parameters. These parameters were estimated from local disruption events collected from the same sets of myoblasts (red), myotubes (blue) and ATP depleted myoblasts (black) as in Fig. 3. Semi-log representations of (**a**) maximal released energy $\widehat{{E}_{d}}$ from each FIC and (**b**) sum Σ*E*_*d*_/*L* of the released energy normalized to the FIC length from each FIC. (**c**,**d**) Same as (**a**,**b**) in a logarithmic representation. Semi-log representation of (**e**) force drop *F*_*d*_, reconstructed from the whole set of rupture events, and (**f**) the corresponding released energy. (**g**,**h**) Same as (**e**,**f**) in a logarithmic representation.

The reconstruction of the histograms of the rupture event maximal energy $\widehat{{E}_{d}}$ and of the sum of the energies Σ*E*_*d*_/*L* rescaled by the length of the FICs for the three sets of FICs (Fig. 8) confirms that the myotubes behaved quite differently than myoblasts (GM and ATP depleted). The distributions of $\widehat{{E}_{d}}$ and Σ*E*_*d*_/*L* are found very similar for normal and ATP depleted myoblasts, even though these later ones have lower *G*_*g*_ and *G*_*i*_ values (Fig. 3(a,b)); this relative weakness of the ATP depleted myoblasts seems to be compensated by a greater dissipative loss (Fig. 3(c)). Our results further show that the myotubes could not sustain very large amplitude energy events. To obtain a more complete picture, we plotted the histograms of the force drop and energy of all the rupture events, independently of the FICs from which they were extracted (Fig. 8(e–h)). Both the distributions of ${\mathrm{log}}_{10}({F}_{d})$ (Fig. 8(e)) and ${\mathrm{log}}_{10}({E}_{d})$ (Fig. 8(f)) for myotube rupture events are rather symmetric and single hump shaped, as an indication that these cells experience failure events of the same nature. In contrast, the distributions of ${\mathrm{log}}_{10}({F}_{d})$ and ${\mathrm{log}}_{10}({E}_{d})$ for myoblasts (normal and ATP depleted) are not single humped but rather spread over larger intervals of values, reflecting the possible mixture of two types of failure events: the lower released energy (ductile) ones, similar to those of myotubes, and the higher released energy (brittle) events typical of myoblasts. The presence of these higher energy failure events might explain why the corresponding FICs had higher dissipative loss *D*_*l*_ (Fig. 3(c)). Those cells which were able to develop higher local stresses (and brittle failures) were also recognized as strain-hardening with *W*_*g*_/*W*_*i*_ > 1 (Fig. 3(d)).

## Discussion

Comparing the different morphologies of myoblasts and myotubes and more precisely their actin CSK networks from fluorescence microscopy images has helped us unravel the origin of the two groups of failure events detected in this study (Fig. 1 and Supplementary Figs S1–S3). One major characteristic of the actin CSK of adherent myoblasts relies on the bundles of F-actin that form thick and stiff stress fibers (perinuclear stress fibers) above the nucleus to maintain some pressure on it and to facilitate a strong internal activity involving DNA and chromatin during *G*_1_, *S* and *G*_2_ phases. The myotubes are differentiated cells that no longer divide and these perinuclear tensed stress fibers are not needed. When indenting myoblasts, we have observed that the cell stiffness was reinforced upon indentation (strain-stiffening), as the signature of a possible local reinforcement of the perinuclear zones by actin stress fibers. Another important concomitance was the fact that those strain-stiffening cells were also those with higher energy disruption events and higher dissipative loss. These disruption events could then be directly related to local brittle failures of the actin stress fibers. Conversely, these high energy failures almost vanished for myotubes because these differentiated cells have a very different actin network organization. These multinucleated elongated cells were obtained by the fusion of bipolar elongated shape myoblasts (plasma membrane fusion). This required not only non-muscle myosin II and actin dynamics but also cadherins, integrins and DOCK1 (dedicator of cytokinesis protein 1, previously known as DOCK180), all *via* the activation of Rac1^73,74^. Typical bipolar-shaped cells committed to membrane fusion and thus to myogenic differentiation are pointed out by arrows in Fig. 1(b) (Bottom) and in Supplementary Fig. S3.

With the considered velocity of the AFM tip (1 *μ*m/s), the cell indentation takes only a few seconds (3–4 s) which is at least one order of magnitude shorter than the relevant time scales for nonmuscle myosin II actin CSK remodeling in coordination with the two other CSK networks. To check whether actomyosin dynamics may have a role in these FIC disruption events and to which extent they may impact the systematic loss of mechanical work (*D*_*l*_) recorded upon indentation of living myoblasts, we inhibited ATP synthesis. The mechanical response of ATP depleted cells revealed that they were globally softer but that they dissipated more mechanical work, with a greater propensity to strain-stiffening (〈*W*_*g*_/*W*_*i*_〉 > 3). We were puzzled to observe that the distributions of the force drop and energy values of their FIC disruption events were very similar to normal myoblasts, as if these cells would have kept locally the actin network cross-linked architecture. ATP depletion affects dramatically the actin network during the early hours after drug administration^69^. F-actin depolymerizes after ~30 minutes, before apical actin ring loss, and actin aggregates appear in the perinuclear regions after ~60 minutes^69^. Disruption of the well-organized F-actin network (stress fibers and actin cortex) and secondary formation of ectopic actin aggregates (especially in perinuclear regions) were observed in different cell lines after ATP depletion^67,68,75^. Microtubules have a persistence length ranging from 5 to 100 *μ*m *in vitro*, which makes them substantially stiffer than other cytoskeletal filaments (when considered as isolated filamentous structures). High resolution microscopy techniques have highlighted the existence of smaller persistence length filaments that would be due to non-thermal force bending, suggesting the interplay of forces exerted by active molecular motors or passive cross-linkers of the actin CSK network and/or the actin cell cortex^48,76^. ATP depletion does not impact the microtubule polymerization which is GTP dependent, these filaments remain intact, and the microtubule CSK network is unchanged. We observed that both *G*_*g*_ and *G*_*i*_ of the C2C12 myoblasts decrease upon ATP depletion (Table 1), which seems to indicate that the actin stress fiber tenseness is one of the major ingredient for the cell elasticity.

We have to remember that ATP depletion does not detach non muscle myosin II from actin filaments but rather “freezes” the actomyosin networked CSK (passive cross-linking by ADP Non-Muscle Myosin II (ADPNMMII)) in a jelly state reminiscent of the initial spread adherent morphology^66^. This local freezing favours the aggregation of small and punctuate actin particles which may act as additional obstacles for the cantilever tip penetration (leading to brittle failures of the network). Finally, the fact that blebbistatin treated myoblasts behaved much softer and less dissipative than the three other tested examples of muscle cells, with quite undetectable disruption events, was an additional indication of the presence of tightly cross-linked structures in the myoblast actin CSK (stress fibers for normal myoblasts and (ADPNMMII-actin aggregates for ATP depleted myoblasts). Fluorescence microscopy images of blebbistatin treated myoblasts provided a visual confirmation of their complete loss of mechanical actomyosin contractility with no evidence of perinuclear stress fibers (Supplementary Fig. S2).

To conclude, we proposed in this study an original method for analyzing the temporal response of living muscle precursor cells upon sharp shearing indentation, and quantifying their aptitude to sustain such a local stress. Rupture events encountered during the force-indentation curves were related to local disruptions of actin cytoskeleton structures, the strongest ones being produced by the tighter and stiffer structures (stress fibers or actin aggregates). This local malleability and susceptibility to actin CSK failures are an important aspect of living cell dynamics, shape maintenance and quick recovery from local stresses. They also play a major role in their quick adaptation to various external environments, such as the differentiation of myoblasts and their fusion into myotubes for example.

## Methods

### Cell culture and differentiation

#### C2C12 myoblast culture

C2C12 myoblasts (ATCC CRL-1772) were cultured in a growth medium (GM) composed of high glucose (4,5 g/L) Dulbecco’s Modified Eagle Medium with L-glutamine (DMEM, PAA, GE Healthcare) supplemented with 20% fetal bovine serum (FBS, PAA), 1% penicillin-streptomycin antibiotics (100 U/ml penicillin and 100 *μ*g/ml streptomycin, Gibco, Thermo Fisher Scientific) and 10 mM HEPES (Gibco). GM was stored at 4 °C up to 1 month; fresh GM was replaced every 2 days. The myoblasts were maintained in a 5% CO_2_ atmosphere at 37 °C inside 90 mm diameter petri dishes until ~70% confluency. To avoid that 60–70% confluent myoblasts differentiate spontaneously, the dishes were washed with preheated PBS and cells were detached from the dish bottom with 0.25% trypsin-EDTA (Gibco) for 3 minutes at 37 °C. Then the cells were harvested and either re-plated at lower concentration or frozen-stored. Prior to AFM FIC collection, C2C12 myoblasts (~1.5 10^5^ cells from passages 10 to 14) were seeded on collagen coated petri dishes CCPDs (35 mm diameter) in GM and kept at least 24 hours at 37 °C with 5% CO_2_. Then the medium was changed and replaced by 2 ml of GM and the petri dish was transferred to the AFM. Each sample was used within 2 to 3 hours and discarded afterwards.

#### C2C12 myoblast differentiation

Confluent (60%) C2C12 myoblasts on CCPDs were induced to differentiation, replacing GM by a differentiation medium (DM)^77^. DM was composed of high glucose (4,5 g/L) Dulbecco’s Modified Eagle Medium with L-glutamine (DMEM, PAA), supplemented with 2% Donor Horse Serum (HS, PAA), 1% penicillin-streptomycin antibiotics (100 U/ml penicillin and 100 *μ*g/ml streptomycin, Gibco) and 10 mM HEPES (Gibco). The myoblasts were maintained in DM at 37 °C with 5% CO_2_ for at least 5 days, renewing the medium every 2 days. Different culture supports were used (glass bottom petri dishes, SPDs and CCPDs) to compare their impact on C2C12 cell myogenic differentiation. C2C12 myoblast differentiation was monitored by DIC and time-lapse video recording. Multinucleated myotubes were observed with fluorescence confocal microscopy and probed by AFM at their 5^*th*^ day of differentiation. A fusion index, defined as the percentage of nuclei contained in myotubes compared to the total number of nuclei observed in each image field, was used to quantify the level of myotube formation in different culture conditions.

#### C2C12 myoblast fixation for fluorescence microscopy

C2C12 myoblasts grown on CCPDs (35 mm diameter) were rinsed twice with preheated PBS and fixed with freshly made 4% paraformaldehyde (PFA, Fluka, St. Louis, MO) in PBS for 20 minutes at room temperature (RT) (24 °C). Then the sample was maintained in 2 ml PBS before AFM measurements which lasted ~4 hours at RT.

#### ATP depletion

Living cells rely on a combination of oxidative and glycolytic energy metabolism for ATP production. For a complete ATP depletion, both pathways must be inhibited^78^, namely the mitochondrial electron transport chain (ETC) complex III with antimycin A (AMA)^79^ and glycolysis with 2-deoxy-D-glucose (2-DG)^80^. ATP depletion buffer composition: 140 mM NaCl, 5 mM KCl, 1 mM MgCl_2_, 2 mM CaCl_2_, 10 mM HEPES, 6 mM 2-DG (Sigma), 5 *μ*M AMA (Sigma). The filtered solution was adjusted to pH 7.4 and stored at −20 °C. CCPDs with adherent C2C12 myoblasts were first rinsed twice with preheated PBS and filled with freshly thawed ATP depletion buffer (2 ml). 15 minutes were necessary for the cellular ATP concentration to decrease below 7% of its initial concentration^81^. AFM FIC capture was performed on single cells, inside the ATP-depletion buffer at RT for 2–3 hours, before discarding the CCPD.

#### Blebbistatin treatment

C2C12 myoblasts were treated with (S)-(-)-Blebbistatin (Santa Cruz Biotechnology) to inhibit the activity of the non-muscle myosin II^82^. Aliquots of 100 *μ*M blebbistatin dissolved in dimethylsufoxide solution (1% DMSO in GM) were stored at −20 °C. GM was replaced with the same amount (2 ml) of preheated GM containing blebbistatin (50 *μ*M) for 20 minutes^83^. AFM experiments on C2C12 cells inside GM-blebbistatin were performed in ~2–3 hours at RT.

#### Petri dish surface treatment

We tested different surface treatments: gold coating, standard (SPD) and type I collagen coated petri dishes (CCPD), and we observed that CCPDs lead to greater C2C12 myoblast lengths, widths and areas. In this study, we chose type I collagen for myoblast adhesion because this protein is one of the major insoluble fibrous protein found in the *in vivo* extracellular matrix (ECM). Type I collagen aqueous solution (3 mg/ml) from bovine skin and tendon BioReagent (Sigma Aldrich) was diluted in ultra-pure water to get 100 *μ*g/ml. Petri dishes were incubated three hours with this solution (8 *μ*g/cm^2^) at 37 °C to allow proteins to bind, dried overnight at RT under clean atmosphere and rinsed with Dulbecco’s Phosphate Buffer Saline (PBS, Sigma) before use.

### Mechanical indentation experiments

A CellHesion 200 Atomic Force Microscope (AFM, JPK Instruments) coupled to a transmission inverted microscope and a CCD camera was used for nano-indentation experiments. The apparatus was equipped with a *X*-*Y* Motor Precision Stage (JPK) with 20×20 mm motorised stage, a vibration isolation table (Melles Griot), a foam-based acoustic isolation system and a white light LED illumination (Thorlabs, MCWHLS). The AFM Z-piezotransducer with movement range of 100 *μ*m was controlled by a closed loop feedback system with sub-nanometric precision. Proportional gain (*P* gain) was set at 20 and integral gain (*I* gain) at 0.002. The calibration of the AFM probes was performed via the thermal noise method^84^. The vertical deflection (Δ*D*) (nm) of the cantilever is proportional to the force applied to the sample; it can be converted to a tip-sample interaction force Δ*F* (nN) knowing the stiffness of the cantilever *k* (N/m) through the Hooke’s law: Δ*F* = *k*Δ*D* and the sensitivity of the photodiode quadrant.

Force indentation curves (FICs) were recorded with sharp tip triangular (SNL) or rectangular cantilevers (qp-CONT) (see Supplementary Information) and targeted on perinuclear zones. To evaluate the impact of both the hydrodynamic drag and the damping of adherent cell layers on FIC shapes, four set of FICs were recorded at different scan velocities (typically from 0.1 to 10 *μ*m/s) on two myoblasts and two myotubes. For each speed, 10 FICs were collected with a force set point of 9 ± 1 nN. Between each 10 FIC recording series, the sampling rate was adjusted to collect similar sample size (~20000) for each FIC. To reconstruct the mechanical parameter histograms, a protocol for cell indentation was elaborated: for each cell, 30 successive FICs were recorded on the perinuclear zone. The force set-point, the indentation velocity, the cantilever displacement range Δ*Z* and the sampling rate were fixed to 8 ± 2 nN, 1 *μ*m/s, 6 ± 2 *μ*m and 3.5 kHz respectively.

### Time-frequency analysis of FICs

#### Correcting, filtering and deriving FICs

The first step was to eliminate the drift of the FICs resulting from either a mis-alignment of the laser photodiode beam on the tip of the cantilever or an hydrodynamic drag produced by the liquid surrounding the cantilever. The elimination of the drift coming for optical misalignment was performed from a parametrization of the sum of the loading and unloading curves, out of contact with the sample, assuming that the hydrodynamic drag inflects similarly the loading and the unloading curves. To parametrize and compensate the hydrodynamic drag force *F*_*d*_, the difference of the out-of-contact loading and unloading curves (*Z* − *Z*_*c*_ < −500 nm) corresponding to 2*F*_*d*_ was used. The second step was to filter the FICs to get rid of the background noise and, when needed, to compute the successive derivatives of the FICs. These two tasks were achieved simultaneously using the continuous wavelet transform^85–89^. Details can be found in the Supplementary Information.

#### Integral representation of force-indentation curves

Viscoelasticity theories developed in the second half of the twentieth century^90,91^ have led to general hereditary integral representation of stress-strain relationships for the indentation of linear viscoelastic materials by axisymmetric indenters:

1 $$F(t)=\frac{4}{1-\nu }{C}_{n}\,{\int }_{0}^{t}\,G(t-\tau )\frac{d{h}^{(n+1)/n}(\tau )}{d\tau }d\tau ,$$

where *G*(*t*) is the stress relaxation modulus, *ν* the Poisson ratio, *θ* the cantilever tip half-angle, *F* is the loading force, *h* = *Z* − *Z*_*c*_ describes the displacement of the indenter, and *n* is a positive integer which depends on the shape of the indenter. The stress relaxation modulus *G*(*t*) retains the memory of the deformation. For a pyramidal indenter tip, we have *n* = 1 and *C*_1_ = tan *θ*/*π*, where *θ* is the nominal tip half-angle:

2 $$F(t)=\frac{4\,\tan \,\theta }{\pi (1-\nu )}\,{\int }_{0}^{t}\,{G}_{r}(t-\tau )\frac{d{h}^{2}(\tau )}{d\tau }d\tau .$$

Since the cantilever is swept at constant velocity *V*_0_, *dZ* = *V*_0_ *dt*, and the stress relaxation modulus *G* can be rewritten as:

3 $$G(Z)=\frac{\pi (1-\nu )}{8\,\tan \,\theta }\frac{{d}^{2}F(Z)}{d{Z}^{2}},$$

meaning that the variation of *G* with *Z* keeps the memory of the whole deformation. For pyramidal (or conical) tips, Eq. (3) establishes that the stress relaxation modulus can be obtained from the second-order derivative of the FIC with respect to *Z*, without assuming *a*-*priori* a particular viscoelastic or plastic cellular model. This approach is therefore quite attractive for living cells which are hardly approximated by a combination of springs and dashpots.

The first- and second-order derivatives of *F*(*Z*) (see Supplementary Eqs (S12) and (S13)) and hence *G*(*Z*) were computed from wavelet transforms of the FICs at a smoothing scale *s*^53,54,92^:

4 $$G(Z)=\frac{\pi (1-\nu )}{8\,\tan \,\theta }{T}_{{g}^{(2)}}[F](Z,s).$$

#### Global mechanical parameter estimation from FICs

If the cells were homogeneous elastic balls, the FICs should be pure parabola^55,91^

5 $$F(Z)=\frac{4\,\tan \,\theta }{\pi (1-\nu )}{G}_{g}{(Z-{Z}_{c})}^{2},$$

the curvature of which is proportional to a global shear relaxation modulus *G*_*g*_. *G*_*g*_ is the prefactor of the parabola that crosses both the contact point and the final set point *Z*_*sp*_ of the FIC (blue curves in Fig. 2(a,b)). If *F*(*Z*) is a parabola, $\sqrt{F(Z)}$ is linear in *Z* (Fig. 2(c,d)). Comparing $\sqrt{F(Z)}$ (red curve) and $\sqrt{\frac{4\,\tan \,\theta }{\pi \mathrm{(1}-\nu )}{G}_{g}}(Z-{Z}_{c})$ (blue curve) tells us if the cell sustains the same modulus *G* during its deformation. Fitting $\sqrt{F(Z)}$ on the first 500 nm after contact gives an approximate initial local cell elasticity that we note *G*_*i*_. We use *G*_*g*_ and *G*_*i*_ as bounds for the cell stiffness. The shear modulus *G* of the myoblast in Fig. 2(c) is bounded from above by *G*_*i*_ and from below by *G*_*g*_; this behavior is typical of nonlinear strain-softening materials and marks the inability of the cell to maintain a high rigidity upon deformation. Inversely, the shear modulus *G* of the myoblast in Fig. 2(d) is bounded from above by *G*_*g*_ and from below by *G*_*i*_, typical of nonlinear strain-hardening materials. In that case, the cell behaves as quite soft upon contact and its rigidity increases upon deformation. Actually this classification, proper to mechanical engineering and rheology, is not strictly correct in the context of living cells, not only because cells are made of sub-compartements with distinct mechanical properties but also because they are active systems. Given the temporal duration of the FICs (a few seconds only) in this study, we were rather sensing spatial and gradual variations of the cell rigidity when getting closer to the nucleus.

Loading and unloading FICs collected from living cells are rarely superimposed, reflecting that a fraction of the input work *W*_*l*_ (loading FIC) is not recovered in the output work *W*_*u*_ upon strain release (unloading FIC)^93,94^. This dissipation of mechanical work is described by the ratio *D*_*l*_:

6 $${D}_{l}=\frac{{W}_{l}-{W}_{u}}{{W}_{l}}=\frac{{\int }_{{Z}_{c}}^{{Z}_{sp}}\,{F}_{l}dZ+{\int }_{{Z}_{sp}}^{{Z}_{c}}\,{F}_{u}dZ}{{\int }_{{Z}_{c}}^{{Z}_{sp}}\,{F}_{l}dZ}.$$

*D*_*l*_ tends to zero for a purely elastic material and to 1 for a purely viscous material. The work integrals corresponding to the parabolic interpolations *G*_*g*_ and *G*_*i*_ are also computed accordingly:

7 $${W}_{g}={\int }_{{Z}_{c}}^{{Z}_{sp}}\,\frac{4\,\tan \,\theta }{\pi (1-\nu )}{G}_{g}{(Z-{Z}_{c})}^{2}\,dZ,\,{\rm{and}}\,{W}_{i}={\int }_{{Z}_{c}}^{{Z}_{sp}}\,\frac{4\,\tan \,\theta }{\pi (1-\nu )}{G}_{i}{(Z-{Z}_{c})}^{2}\,dZ.$$

The temporal evolution of the different work integrals (*W*_*l*_, *W*_*u*_, *W*_*g*_ and *W*_*i*_) during 30 successive load-unload scans are compared in Fig. 2(e,f). The myoblast of Fig. 2(e) that we have already considered as strain-softening has its ratios *W*_*l*_/*W*_*g*_ > 1 and *W*_*l*_/*W*_*i*_ < 1; they both never cross the value 1 during the 30 successive load-unload scans. When these two ratios collapse to 1, a pure elastic ball response is found, this occurs for FIC index = 4 only in this example. We also note that while repeating the cell deformation (after 20 load-unload scans), the ratios tend to be more distant from 1 than initially, meaning that the cell response gets farther from a simple elastic ball with time. For the myoblast of Fig. 2(f), the situation is inverted, *W*_*l*_/*W*_*g*_ < 1 and *W*_*l*_/*W*_*i*_ > 1, and as before these two ratio curves do not cross during the 30 load-unload scans. Surprisingly, whereas *W*_*l*_/*W*_*g*_ is quite constant, *W*_*l*_/*W*_*i*_ is very irregular and increases to values around 3 after 8 load-unload scans. This evolution can be explained by the fact that, underneath a softer cell cortex, these indentations progressively unveil a stiffer cell subdomain that ultimately yields under the constraint (30th value). The dissipation loss *D*_*l*_ is only slightly smaller for the second myoblast (Fig. 2(f)) than for the first one (Fig. 2(e)), meaning that cells classified as strain-hardening would be less dissipative that strain-softening ones. In both cases, *D*_*l*_ did not change much during the 30 successive indentations. If the loading and unloading FICs could be interpolated with simple power laws:

8 $${F}_{l}={A}_{l}{(Z-{Z}_{c})}^{{\alpha }_{l}},\,{F}_{u}={B}_{u}{(Z-{Z}_{c})}^{{\beta }_{u}}\,{\rm{with}}\,{\beta }_{u} > {\alpha }_{l},$$

then, given that *F*_*i*_(*Z*_*sp*_) = *F*_*u*_(*Z*_*sp*_), *D*_*l*_ can be simply expressed as a function of the ratio of the exponents *α*_*l*_ and *β*_*u*_:

9 $${D}_{l}=1-\frac{{\alpha }_{l}}{{\beta }_{u}}.$$

We thus conclude that if *D*_*l*_ does not change markedly while *W*_*l*_/*W*_*i*_ is multiplied by 3 (Fig. 2(f)), both (nonlinearity) exponents *α*_*l*_ and *β*_*u*_ must vary consistently with the indentation *Z*.

Figure 5 shows two other myoblasts with amazing mechanical responses. In both cases, the global parabola *G*_*g*_ provides a rather good approximation of the general FIC trend. However, we note that *G*_*i*_ (Fig. 5(c,d)) and the ratio *W*_*l*_/*W*_*i*_ are more erratic than in the previous cases (Fig. 2), and that they moreover cross the line 1. In that case, the strong irregularity of these work integral ratios is also accompanied by visible disruptions of the FICs, corresponding to successive local hardening and softening of the cells. In Fig. 5(a,c), we can identify at least four FIC disruption events at $Z-{Z}_{c}\sim 1.4,1.8,2.3$ and 3.1 *μ*m. These disruptions make the determination of *G*_*i*_ from $\sqrt{F(Z)}$ more sensitive to the size of the parametrization interval in *Z* and in turn explain the irregularity of *W*_*l*_/*W*_*i*_.

#### Tracking the rupture events in FICs

The protocol that we have elaborated to track singular events in FICs is shown in Fig. 6. This protocol relies on a space-scale decomposition of the FICs and of their successive derivatives, using the continuous wavelet transform^85–89^. More details on this methology can be found in the Supplementary Information. We quantitatively tested both the second- and third-order derivatives of the FICs and we reached the conclusion that the most efficient tool was the second-order derivative of the FICs, computed with a second-order derivative of a Gaussian wavelet of size $w=2\sqrt{2}s=3.86$ nm (Eq. (4)). Noticing that the FIC disruption events occurred in between two consecutive minima *G*_*m*_ and maxima *G*_*M*_ of *d*^2^*F*(*Z*)/*dZ*^2^, we took the local minima *G*_*m*_ as searching criteria (Fig. 6(d)). We defined a threshold |*G*_*m*_| from the distribution of *G*_*m*_ values computed on a representative set of FICs, to discriminate the disruption events from the background noise. The prominence of these negative peaks was set to |*G*_*m*_| ≥ 5 MPa. In the right neighbourhood of these peaks, we searched for a local maxima of *d*^2^*F*(*Z*)/*dZ*^2^ with a peak prominence ≥1 MPa. *G*_*m*_ and *G*_*M*_ are marked with black symbols in Fig. 6(c,d). From the two positions of *G*_*m*_ and *G*_*M*_, we could then detect the beginning and the end of the disruption events, represented with blue symbols in Fig. 6(a,b). The distance between these two positions is noted Δ*Z*_*d*_. Finally, the force drop *F*_*d*_ was corrected, taking into account the increasing trend of the FIC after the rupture event. A linear interpolation of the FIC in a small interval (~20 nm) beyond the local minima of the FIC gave the best interpolation. Nonlinear interpolations of the FIC did not work better. We defined the energy released during this disruption event as:

10 $${E}_{d}={F}_{d}\,{\rm{\Delta }}{Z}_{d}.$$

## Electronic supplementary material

Supplementary information file
