# Supplementary material for: Prestressed cells are prone to cytoskeleton failures under localized shear strain: an experimental demonstration on muscle precursor cells
Source: Sci Rep. 2018 Jun 5;8:8602. doi: 10.1038/s41598-018-26797-4 (PMC5988700; doi:10.1038/s41598-018-26797-4)
Supplement: Supplementary file 1 — Supplementary information file [file 41598_2018_26797_MOESM1_ESM.pdf]

Supplementary Information

Prestressed cells are prone to cytoskeleton failures under localized shear strain: an experimental demonstration on muscle precursor cells

Laura Streppa[1,2,3], Francesca Ratti[2,3], Evelyne Goillot[2,3], Anne Devin[4], Laurent Schaeffer[2,3], Alain Arneodo[1,2,5] and Françoise Argoul[1,2,5,*]

[1] Ecole Normale Supérieure de Lyon, CNRS, Laboratoire de Physique, UMR5672, F-69007 Lyon, France
[2] Université de Lyon 1, F-69100 Villeurbanne, France
[3] Ecole Normale Supérieure de Lyon, CNRS, LBMC, UMR5239, F-69007 Lyon, France
[4] Université de Bordeaux, CNRS, IBGC, UMR5095, F-33077 Bordeaux, France
[5] Université de Bordeaux, CNRS, LOMA, UMR5798, F-33405 Talence, France
[*] corresponding author: francoise.argoul@u-bordeaux.fr

Methods - Complements

Fluorescence microscopy and staining

Fluorescence confocal microscopy imaging was performed with an inverted scanning confocal microscope (ZEISS LSM710) equipped with a motorized X-Y stage and a set of ZEISS objectives with magnification from 10X to 63X. Fluorescent images (1224 x 900 pixels) were collected using a 405 nm blue-violet laser diode (DAPI, AMCA), a 488 nm argon laser (Alexa Fluor 488), and a 561 nm diode-pumped solid state (DPSS) laser (Cy3). The excitation and the fluorescence signal acquisition of each fluorophore was executed in sequential multi-tracking channels in order to avoid bleed-through crossover artefacts. Saturation was checked and avoided using range indicators of the Zen software. The scan speed and image averaging were optimized before image acquisition in order to have the highest signal-to-noise ratio for a minimum cost of time. Z-Stack images of 9 μm $\pm$ 3 μm depth with 0.5 μm interval between each slice were acquired with the Zen software. ImageJ 1.47v processing program was used to analyze the confocal microscopy images and to reconstruct the Z projections.

Fluorescent labelling of specific intracellular structure of C2C12 cells was performed combining immunofluorescence and non-antibody labelling techniques. Adherent C2C12 myoblasts ($\sim 10^5$) and confluent myotubes on 35 mm diameter CCPDs were rinsed twice with preheated PBS and fixed with freshly made 4% paraformaldehyde (PFA, Fluka) in PBS for 20 minutes at room temperature (RT). The samples were then permeabilized with 0.05 % Triton X-100 (Euromedex, Souffelweyersheim, France) in PBS for 10 minutes at RT before being saturated with 1 % bovine serum albumins (BSA, Sigma) in PBS and kept 30 minutes at RT. The samples were stained either for F-actin, β-tubulin and nucleus, or for the three cytoskeleton filaments according to the two following protocols. In the fist case, after incubation with mouse monoclonal Cy3-conjugate anti-β-tubulin (Sigma) diluted 1:200 in 1 % BSA PBS for 1 hour at RT in a humid and dark chamber, the cells were incubated with phalloidin-Alexa Fluor 488 (Molecular Probes) diluted 1:100 in 1 % BSA PBS for 15 minutes at RT in a humid and dark chamber. Finally, the samples were sealed with glass cover-slides and VectaShield mounting medium (Vector Laboratories) with DAPI (4',6-diamino-2- phenylindole) and stored at least for 10 minutes at 4℃. In the second case before β-tubulin staining, the cells were incubated with mouse monoclonal anti-desmin (DAKO, Agilent Technologies) diluted 1:200 in 1 % BSA PBS over night in humid chamber at 4℃. The day after, incubation with AMCA-conjugated goat secondary antibody anti mouse (Jackson ImmunoResearch) diluted 1:1000 in 1% BSA PBS was carried out for 1 hour at RT in a humid and dark chamber. Then the labelling of β-tubulin and F-actin was performed similarly to previous protocol before sealing the samples with glass cover-slides and VectaShield mounting medium without DAPI and storing them at least 10 minutes at 4℃. In both protocols the samples were gently rinsed with PBS 3 times for 5 minutes between each step. Fluorescence images of C2C12 myoblasts and myotubes in different conditions are shown in Figs 1(a), S1, S2 and S3.

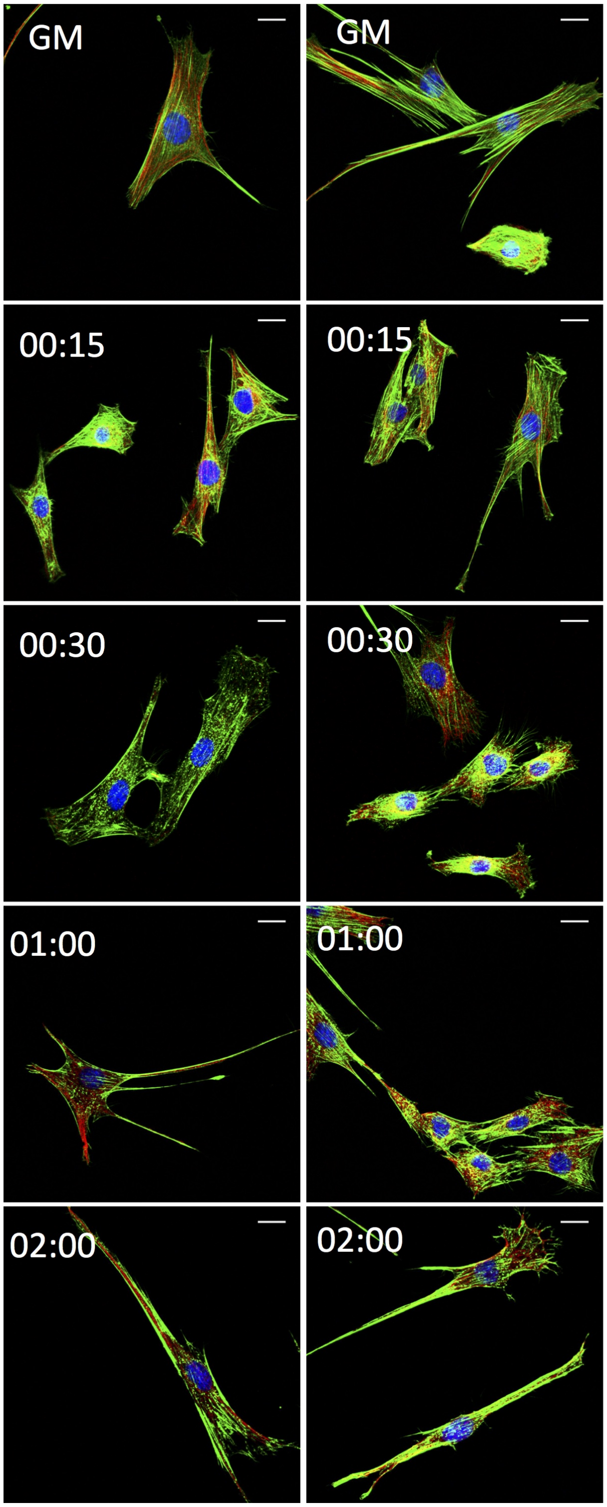

Figure S1. Immuno-fluorescence confocal images of ATP depleted C2C12 myoblasts. C2C12 myoblasts on CCPD were stained at different times after being immersed in the ATP depletion buffer. The samples were kept at room temperature during the treatment. Staining: F-actin (phalloidin-Alexa Fluor 488, green), β-tubulin (anti-β-tubulin Cy3-conjugate, red) and nucleus (DAPI, blue). The time is reported as hh:min. Scale bars: 20 μm.

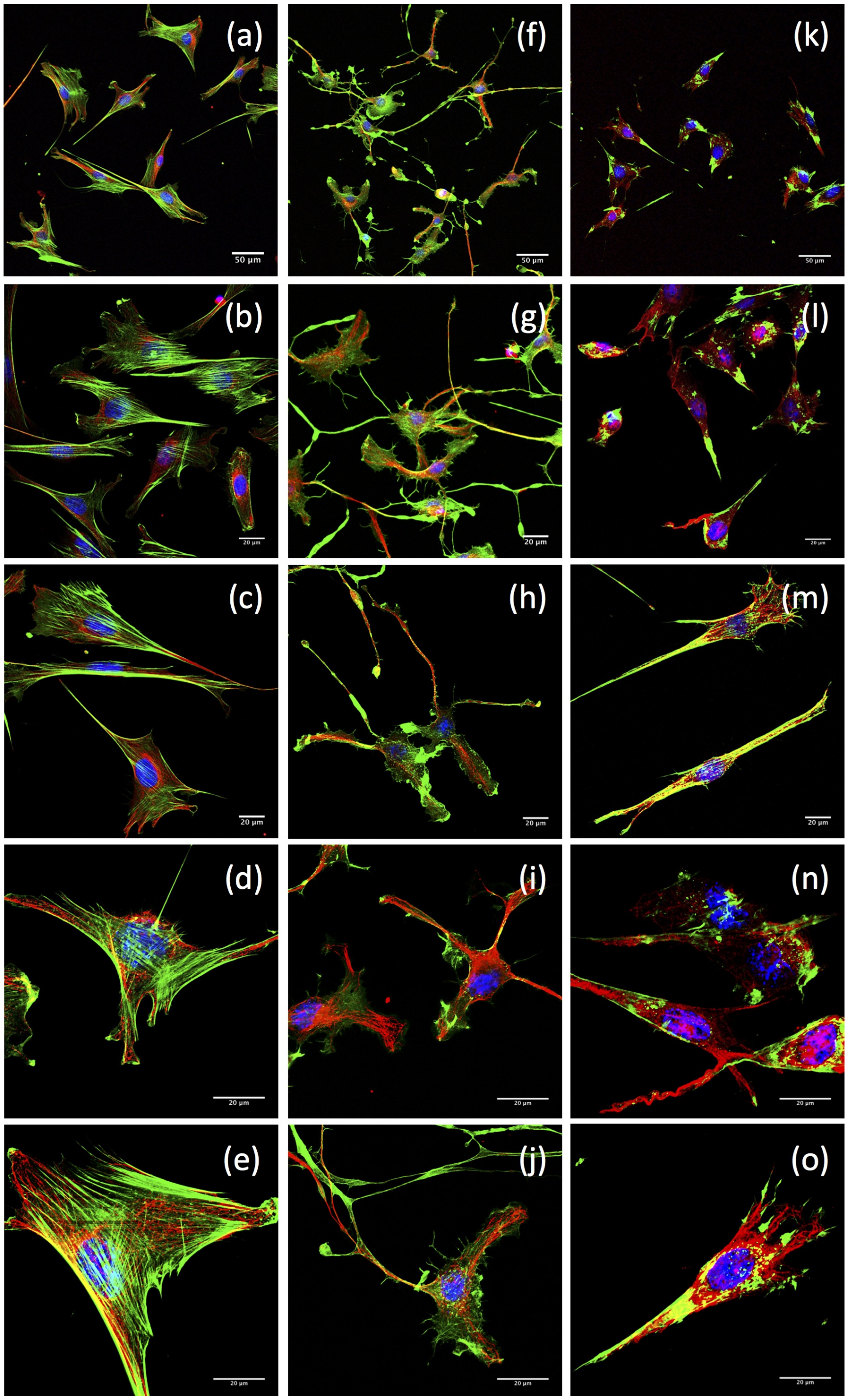

Figure S2. Immuno-fluorescence confocal images of normal, blebbistatin treated and ATP depleted C2C12 myoblasts. C2C12 myoblasts on CCPD were stained after 2h in (a-e) normal GM, (f-j) GM with blebbistatin, and (k-o) in the ATP depletion buffer. The samples were kept at room temperature during the treatments, similar to the AFM experimental conditions. Staining: F-actin (phalloidin-Alexa Fluor 488, green), β-tubulin (anti-β-tubulin Cy3-conjugate, red) and nucleus (DAPI, blue). Scale bars: (a, f, k) 50 μm, all the other images 20 μm.

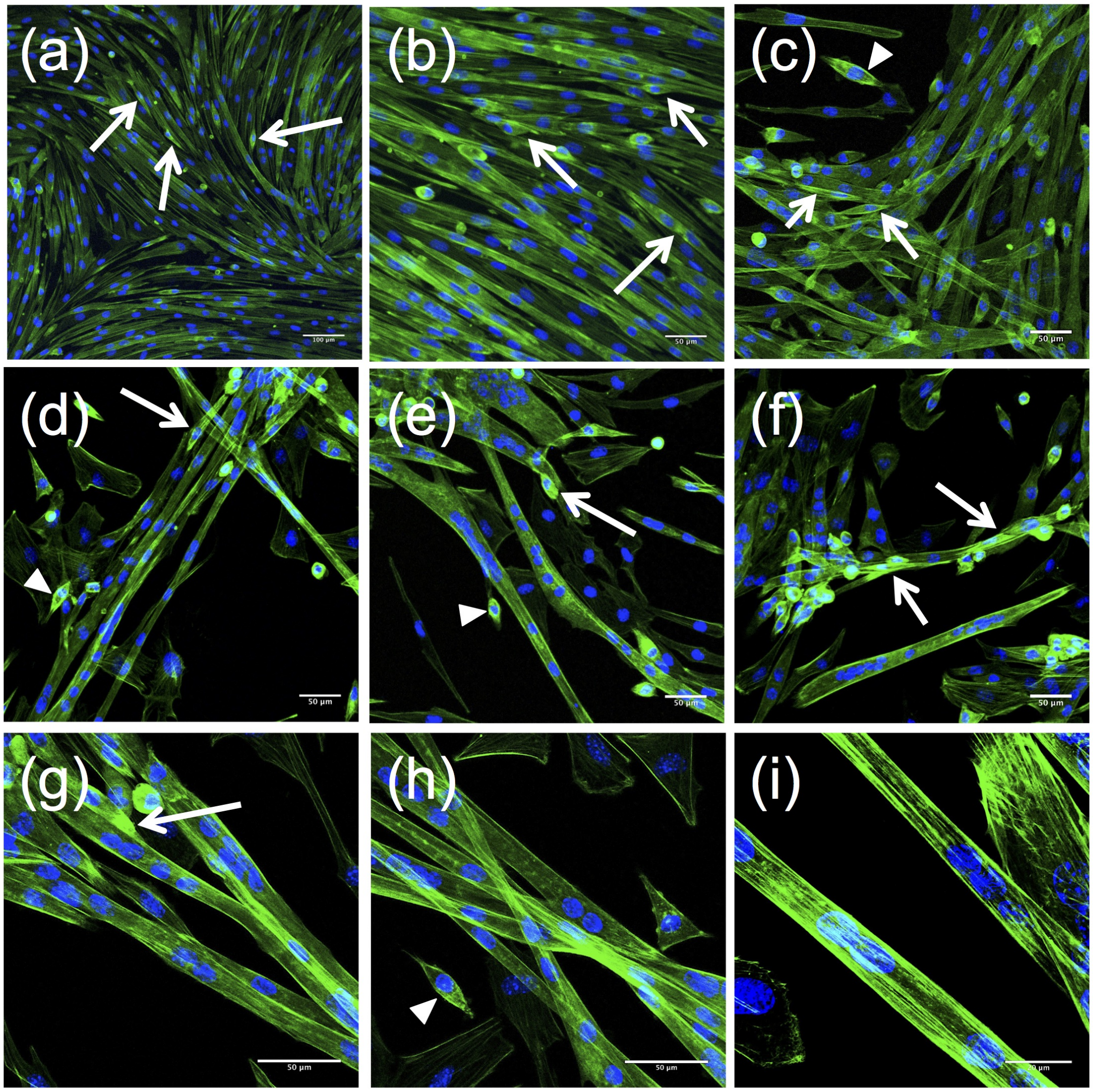

Figure S3. Fluorescence staining of F-actin and nuclei on C2C12 myotubes. Confocal microscopy imaging of MFs (green) and nuclei (blue) of C2C12 myotubes, the fifth day after differentiation induction. White arrows indicate cell fusion events leading to myotubes; arrowheads indicate the typical bipolar shape of myogenic committed myoblasts. Staining: F-actin (phalloidin-Alexa Fluor 488, green) and nuclei (DAPI, blue). Scale bars: (a) 100 μm, (b-h) 50 μm, and (i) 20 μm.

Mechanical indentation experiments

AFM probes and their calibration

We used two types of AFM probes: (i) triangular gold-coated silicon nitride cantilevers from Sharp Nitride Lever (SNL-10, Bruker, Camarillo, CA) with nominal spring constant of 0.06 N/m (min= 0.03 N/m; max= 0.12 N/m) and typical resonant frequency in air: 18 ± 6 kHz; (ii) rectangular partially gold-coated quartz cantilevers (qp-CONT 20, Nanosensors, Neuchatel, Switzerland) with nominal spring constant of 0.1 N/m (min = 0.08 N/m; max= 0.15 N/m) and typical resonant frequency in air: 30 ± 4 kHz. According to the manufacturers, the SNL-10 cantilever tips (resp. qp-CONT 20 cantilever tips) have the following specifications: front angle (FA) 15±2.5°, back angle (BA) 25±2.5° and side angle (SA) 22.5±2.5°(resp. FA=BA=SA=15 ±3°). Before each experiment, cantilever calibration was carefully performed both in air, to verify the correct positioning of the probe and the proper system alignment, and in liquid to estimate the cantilever spring constant (k). First, the deflection sensitivity (nm/V) of the cantilever-photodiode system was evaluated using the in-contact part of force indentation curves (FICs) on a clean glass surface (5 FICs collected in both air and liquid). Then the cantilever spring constant was estimated in air and in liquid by the thermal noise method[1–4] as shown in Fig. S4. From the thermal fluctuations of the cantilever, we can get an

estimation of its spring constant k, assuming energy equipartition and harmonic oscillations[1]:

$$k = \frac{k_B T}{\langle d^2 \rangle} , \quad \text{(S1)}$$

where $\langle d^2 \rangle$ is the mean square displacement of the cantilever from its neutral position, T the absolute temperature in Kelvin and k_B the Boltzmann's constant. Since AFM cantilevers have different geometries and several vibration modes, a correction factor β_n must be included in Eq. (S1):

$$k = \frac{k_B T}{\langle d^2 \rangle} \beta_n , \quad \text{(S2)}$$

where the factor β_n varies according to the vibration mode and the cantilever geometry[4,5]. The value $\beta_n = 0.817$ (resp. 0.778) was used for rectangular (resp. triangular) cantilevers.

Power spectral density (PSD) was computed from AFM cantilever deflection signals captured 100 μm away from the sample surface. Fig. S4(b, e) shows peaks of the two first modes of a triangular AFM cantilever (SNL-10 Bruker) in air and in liquid. Finally, the vertical deflection (ΔD) (nm) of the cantilever being proportional to the force applied to the sample, we converted it to a tip-sample interaction force ΔF (nN) knowing the stiffness of the cantilever k (N/m) through the Hooke's law: $\Delta F = k \Delta D$ and the sensitivity of the photodiode quadrant.

A key issue in the analysis of AFM FICs is the determination of the scan position Z_c where the cantilever tip comes in contact with the tested material, such as a living cell for instance. This quantity is used in particular as a lower bound for the work integrals (Eq. (7)) and as an upper bound for hydrodynamic drag correction. To master this issue, we used a wavelet transform decomposition of the FICs (see next section). For a given width s of the analyzing wavelets $g^{(0)}$ and $g^{(1)}$, we computed the smoothed force $T_{g^{(0)}}[F]$ and its derivative $T_{g^{(1)}}[F]$, and we estimated the contact position Z_c where these two functions cross a given threshold. This threshold was estimated from different smoothing scales values s_g, as a compromise between the need of keeping the transition from out-of-contact to in-contact as sharp as possible in the force derivative $T_{g^{(1)}}[F]$ and the necessity of smoothing the background noise. For this purpose, we used a lower threshold of 5 10^{-5} nN/nm with a $g^{(1)}$ wavelet size of $2\sqrt{2} s_{g^{(0)}} = 110$ nm.

Cantilever spring constant correction

When the force derivative is no longer negligible as compared to the cantilever spring constant k, the FICs must be corrected according to the following equation[6]:

$$Z - Z_c = h + F/k . \quad \text{(S3)}$$

The nominal spring constant of the cantilever ($k \in$ [0.04nN/nm to 0.1 nN/nm]) was chosen large enough for the cantilever deflection to be small compared to the cell deformation. Nevertheless, we have subtracted the correcting term from the FICs for energy integral computation. Note that this correcting term does not affect the computation of the second-order derivative of the force.

Time-frequency analysis of FICs

Historical introduction of the wavelet transform

The wavelet transform is a mathematical time-frequency (time-scale) decomposition of signals introduced in the early 1980s[7]. The wavelet transform has been applied to a great variety of situations in physics, physical chemistry, biology, signal and image processing, material engineering, mechanics, economics, epidemics ...[8–14]. Real experimental signals are very often nonstationary (they contain transient components), and when they are complex or singular, they may also involve a rather wide range of frequencies. It also happens that experimental signals display characteristic frequencies that drift in time. Standard Fourier analysis is therefore inadequate in these situations, since it provides only statistical information about the relative contributions of the frequencies involved in the analyzed signal. The possibility to perform simultaneously a temporal and frequency decomposition of a given signal was first proposed by Gabor for the theory of communication[15]. Later on, two distinct approaches (based on different wavelet transforms) were developed in parallel: (i) a continuous wavelet transform (CWT)[7,11] and (ii) a discrete wavelet transform (DWT)[9]. For singular (self-similar or multi-fractal) signals or images, the CWT transform rapidly became a predilection mathematical microscope to perform space-scale analysis and to characterize scale invariance properties. In particular it was used to elaborate a statistical physics formalism of multifractals[10,13,16–19].

During the past 30 years, the CWT was used for biological applications, on both 1D signals and 2D images[12–14]. As far as 1D signals are concerned, the CWT was applied to AFM force curves collected from single living plant cells[20], living

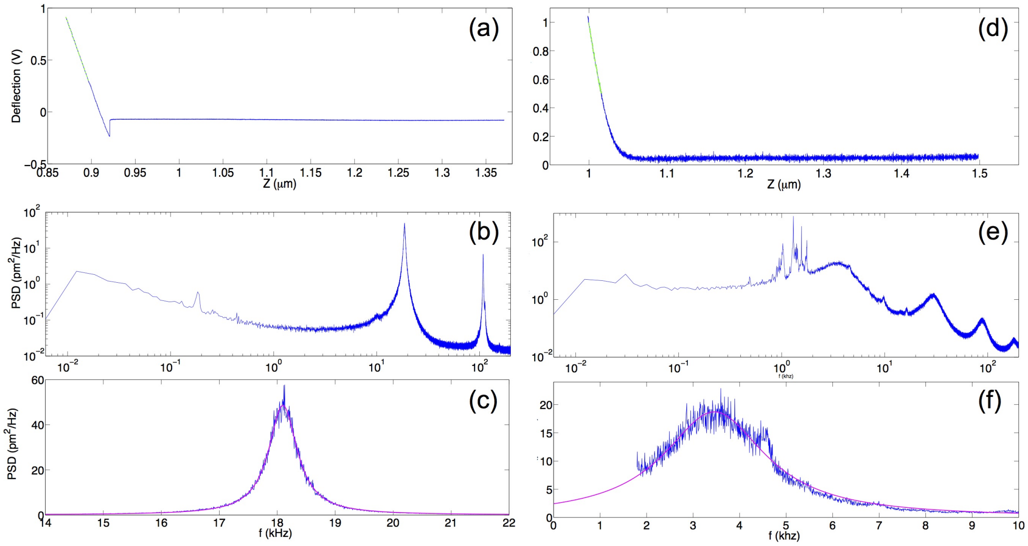

Figure S4. **Illustration of a SNL-10 triangular cantilever calibration**. Calibration of the same AFM probe in air (a-c) and in liquid (d-f). Estimation of the deflection sensitivity of the cantilever using the slope of the loading curves of FICs recorded when indenting (a) a clean glass surface in air and (d) a collagen coated petri dish in growth medium (GM). Power spectral density of the same cantilever thermal (unforced) fluctuations in air (b) and in GM (e). First mode resonance peak (blue) and its fittings with a Lorentzian function (magenta) in air (c) and liquid (f)[4]. Air (resp. liquid) deflection sensitivity: 41.7 nm/V (resp. 32.4 nm/V); first mode resonance frequency: 18.1 kHz (resp. 3.9 kHz); $k = 0.068$ N/m (resp. 0.05 N/m).

hematopoietic stem cells[21,22] and to AFM fluctuation signals to characterize the passive microrheology of living myoblasts[23]. The 1D CWT was also generalized to 2D (and to 3D) CWT[12,13] and it proved again its versatility and power for analyzing AFM topographic images of biosensors[24], fluorescence microscopy images of chromosome territories[25] and diffraction phase microscopy of living cells[26,27]. In the context of this study, we concentrated on 1D Gaussian analyzing wavelets.

Within the norm $\mathscr{L}^1$, the one-dimensional WT of a signal $F(Z)$ reads:

$$W_\psi[F](b,s) = \frac{1}{s}\int_{-\infty}^{\infty} F(Z)\psi^*(\frac{Z-b}{s})dZ\,, \tag{S4}$$

where b is a spatial coordinate (homologous to Z) and s (> 0) a scale parameter. A typical admissible (of null integral) analyzing wavelet $\psi(Z)$ is the second derivative of a Gaussian $g^{(0)}(Z) = e^{-Z^2/2}$, also called the Mexican hat wavelet:

$$g^{(2)}(Z) = -\frac{d^2}{dZ^2}g^{(0)}(Z) = e^{-Z^2/2}(1-Z^2)\,. \tag{S5}$$

Via two integrations by part, it is straightforward to demonstrate that the WT of F with the second derivative of a Gaussian wavelet at scale s, $W_{g^{(2)}}[F](b,s)$, is precisely the second derivative of a smoothed version $W_{g^{(0)}}[F](b,s)$ of F by a Gaussian function at the same scale s:

$$W_{g^{(2)}}[F](b,s) = s^2\frac{d^2}{db^2}W_{g^{(0)}}[F](b,s)\,. \tag{S6}$$

Let us define the first derivative of the Gaussian function:

$$g^{(1)}(Z) = -\frac{d}{dZ}g^{(0)}(Z) = Ze^{-Z^2/2}\,, \tag{S7}$$

and its third derivative:

$$g^{(3)}(Z) = -\frac{d^3}{dZ^3}g^{(0)}(Z) = Z(3-Z^2)e^{-Z^2/2}\,. \tag{S8}$$

With the first and the third derivatives of F, we can write similar relations to Eq. (S6) for the WTs of the odd order derivatives of F[12,14,17]:

$$W_{g^{(1)}}[F](b,s) = s\frac{d}{db}W_{g^{(0)}}[F](b,s)\,, \tag{S9}$$

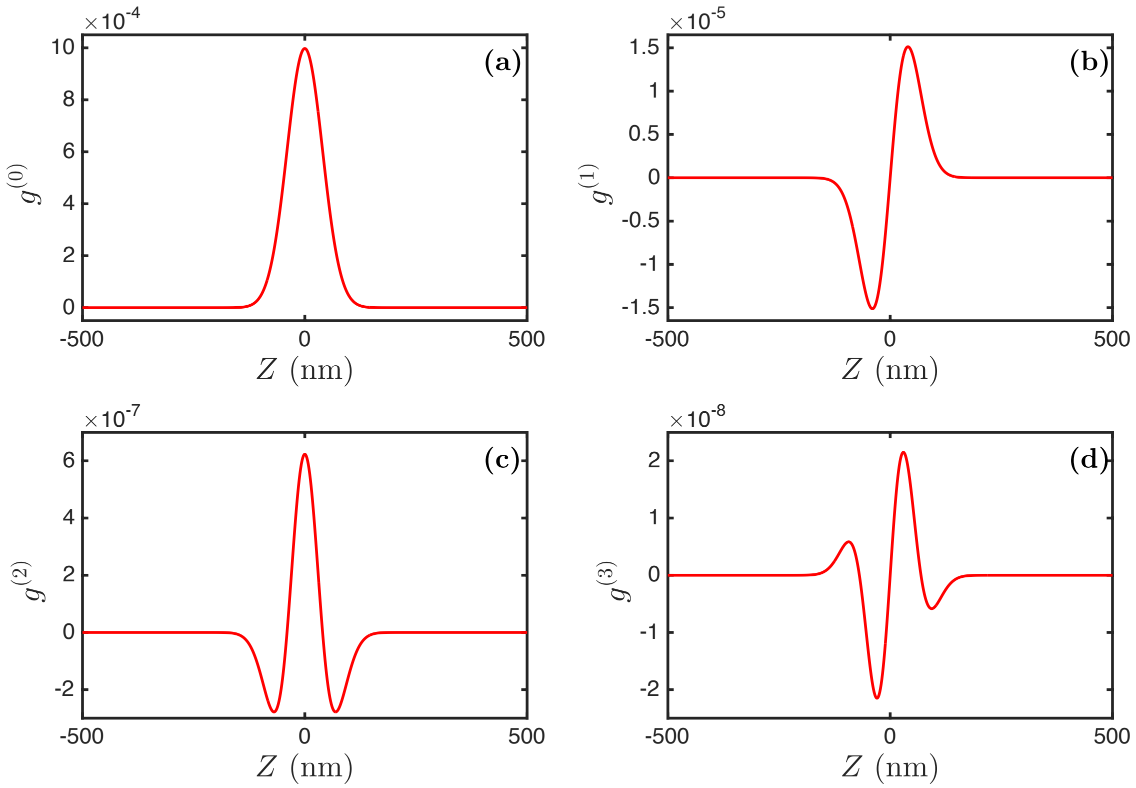

Figure S5. Analyzing wavelets constructed from a Gaussian function. (a) Gaussian function: $g^{(0)}$. (b) First derivative of a Gaussian: $g^{(1)}$ (Eq. (S7)). (c) Second derivative of a Gaussian: $g^{(2)}$ (Eq. (S5)). (d) Third derivative of a Gaussian: $g^{(3)}$ (Eq. (S8)).

and

$$W_{g^{(3)}}[F](b,s) = s^3 \frac{d^3}{db^3} W_{g^{(0)}}[F](b,s) \; . \tag{S10}$$

These four analyzing wavelets are shown in Fig. S5. The zero- and second-order derivatives of the Gaussian ($g^{(0)}$ and $g^{(2)}$) are symmetric functions, the first- and the third-order derivatives ($g^{(1)}$ and $g^{(3)}$) are asymmetric. When we increase the order of derivation, the number of extrema of the wavelet increases from 1 to 4.

Let us point out that the validity of the WT definition (Eq. (S4)) was further extended for distributions including Dirac distributions[17,28]. The interest of the WT method is two-fold. The first advantage is to use the same smoothing function to filter out the experimental background noise and to compute higher-order derivatives (for instance up to third-order in this study) at a well defined smoothing scale s_g. The second advantage relies on the power of the WT to detect local singularities (including rupture events in the FICs) and to quantify their force via the estimate of local Hölder exponents from the behavior across scales of the WT modulus maxima (WTMM)[11–14,17–19,28]. In this study, we used modified versions of the definition (Eq. (S4)) of the WT to get a direct measure of F in nN ($T_{g^{(0)}}[F](b,s)$), dF/dZ in nN/nm ($T_{g^{(1)}}[F](b,s)$), d^2F/dZ^2 in Pascal ($T_{g^{(2)}}[F](b,s)$) and d^3F/dZ^3 in Pascal/nm ($T_{g^{(3)}}[F](b,s)$), once smoothed by a Gaussian window ($g^{(0)}(Z)$) of width s:

$$T_{g^{(0)}}[F](b,s) = W_{g^{(0)}}[F](b,s) \; , \tag{S11}$$

$$T_{g^{(1)}}[F](b,s) = \frac{1}{s} W_{g^{(1)}}[F](b,s) \; , \tag{S12}$$

$$T_{g^{(2)}}[F](b,s) = \frac{1}{s^2} W_{g^{(2)}}[F](b,s) \; , \tag{S13}$$

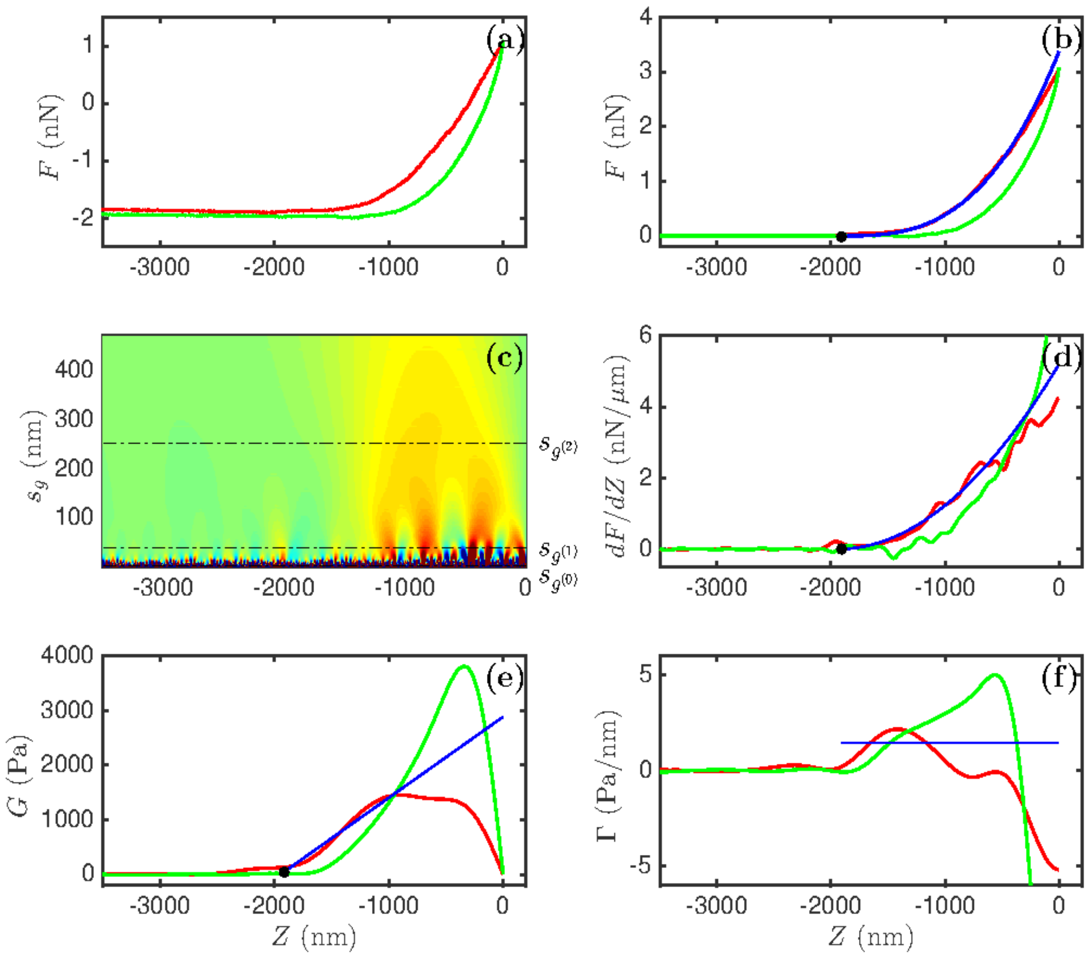

Figure S6. Illustration of the wavelet-based analysis of AFM FICs. (a) Raw FICs recorded from a living myoblast cell (loading curve: red, unloading curve: green). (b) FICs after tilt and hydrodynamic drag corrections. These FICs have also been filtered by a Gaussian of width $2\sqrt{2}s_{g^{(0)}} = 9$ nm. (c) Scale-space representation of the wavelet transform of the loading FIC with the second derivative $g^{(2)}$ of the Gaussian function (Eq. (S5)). The wavelet scales $s_{g^{(0)}}$, $s_{g^{(1)}}$ and $s_{g^{(2)}}$ ($s_{g^{(2)}} = s_{g^{(3)}}$) used for the FIC are outlined with black dashed-dotted horizontal lines. Rainbow color-scale from black to red: [-5 kPa, 5 kPa]. (d) First derivative of the loading FIC, computed with the first derivative $g^{(1)}$ of the Gaussian function (Eq. (S7)) of width $2\sqrt{2}s_{g^{(1)}} = 110$ nm. (e) Second derivative of the loading FIC, computed with the second derivative $g^{(2)}$ of the Gaussian function of width $2\sqrt{2}s_{g^{(2)}} = 710$ nm and expressed in Pa units. (f) Third derivative of the loading FIC, computed with the third derivative $g^{(3)}$ of the Gaussian function (Eq. (S8)) of width $2\sqrt{2}s_{g^{(3)}} = 710$ nm and expressed in Pa/nm units. The blue curves correspond to a nonlinear viscoelastic parametrization of the first derivative of the FIC.

$$T_{g^{(3)}}[F](b,s) = \frac{1}{s^3} W_{g^{(3)}}[F](b,s) \; . \tag{S14}$$

Fig. S6 illustrates on a single FIC (loading and unloading curves) the computation of the wavelet-based force derivatives and their parametrization with nonlinear visco-elastic models. The first derivative of the FIC (Fig. S6(d)) is preferred to the FIC (Fig. S6(b)) for the parametrization because it is more efficient to distinguish the two classes of behaviour (elasto-plastic nonlinear deformation or viscoelastic exponential type relaxation). To perform this systematic classification of FICs numerically, we also computed the second and third derivatives of the FIC (Fig. S6(c,e,f)).

References

1. Sader, J. E., Larson, I., Mulvaney, P. & White, L. R. Method for the calibration of atomic force microscope cantilevers. *Rev. Sci. Instruments* **66**, 3789–3798 (1995).
2. Hutter, J. L. Comment on tilt of atomic force microscope cantilevers: effect on spring constant and adhesion measurements. *Langmuir* **21**, 2630–2632 (2005).
3. Sader, J. E., Hughes, B. D., Sanelli, J. A. & Bieske, E. J. Effect of multiplicative noise on least-squares parameter estimation with applications to the atomic force microscope. *Rev. Sci. Instruments* **83**, 055106 (2012).
4. Schillers, H. *et al.* Standardized nanomechanical atomic force microscopy procedure (SNAP) for measuring soft and biological samples. *Sci. Reports* **7**, 5117 (2017).
5. Stark, R., Drobek, T. & Heckl, W. Thermomechanical noise of a free v-shaped cantilever for atomic-force microscopy. *Ultramicroscopy* **86**, 207–215 (2001).
6. Cappella, B. & Dietler, G. Force-distance curves by atomic force microscopy. *Surf. Sci. Reports* **34**, 1–104 (1999).
7. Grossmann, A. & Morlet, J. Decomposition of Hardy functions into square integrable wavelets of constant shape. *SIAM J. on Math. Analysis* **15**, 723–736 (1984).
8. Meyer, Y. *Wavelets and Applications* (Springer-Verlag, Berlin, DE, 1992).
9. Daubechies, I. *Ten Lectures on Wavelets, vol. 61 of CBMS-NSF Regional Conference Series in Applied Mathematics* (SIAM, Philadelphia, CA, USA, 1992).
10. Arneodo, A., Bacry, E. & Muzy, J. The thermodynamics of fractals revisited with wavelets. *Phys. A* **213**, 232–275 (1995).
11. Mallat, S. *A Wavelet Tour of Signal Processing* (Academic Press, New York, USA, 1998).
12. Arneodo, A., Audit, B., Decoster, N., Muzy, J.-F. & Vaillant, C. Wavelet based multifractal formalism: applications to DNA sequences, satellite images of the cloud structure, and stock market data. In *The Science of Disasters*, 26–102 (Springer Verlag, Berlin, DE, 2002).
13. Arneodo, A., Audit, B., Kestener, P. & Roux, S. Wavelet-based multifractal analysis. *Scholarpedia* **3**, 4103 (2008).
14. Arneodo, A. *et al.* Multi-scale coding of genomic information: From DNA sequence to genome structure and function. *Phys. Reports* **498**, 45–188 (2011).
15. Gabor, D. Theory of communication. *J. Inst. Electr. Eng.* **93**, 429–457 (1946).
16. Bacry, E., Muzy, J. F. & Arneodo, A. Singularity spectrum of fractal signals from wavelet analysis: Exact results. *J. Stat. Phys.* **70**, 635–674 (1993).
17. Muzy, J.-F., Bacry, E. & Arneodo, A. The multifractal formalism revisited with wavelets. *Int. J. Bifurc. Chaos* **4**, 245–302 (1994).
18. Delour, J., Muzy, J. & Arneodo, A. Intermittency of 1d velocity spatial profiles in turbulence: a magnitude cumulant analysis. *Eur. Phys. J. B* **23**, 243–248 (2001).
19. Audit, B., Bacry, E., Muzy, J.-F. & Arneodo, A. Wavelet-based estimators of scaling behavior. *IEEE Transactions on Inf. Theory* **48**, 2938–2954 (2002).
20. Digiuni, S. *et al.* Single cell wall nonlinear mechanics revealed by a multi-scale analysis of AFM force-indentation curves. *Biophys. J.* **108**, 2235–2248 (2015).
21. Laperrousaz, B. *et al.* Revealing stiffening and brittlening of chronic myelogenous leukemia hematopoietic primary cells through their temporal response to shear stress. *Phys. Biol.* **13**, 03LT01 (2016).
22. Laperrousaz, B. *et al.* From elasticity to inelasticity in cancer cell mechanics: A loss of scale-invariance. *AIP Conf. Proc.* **1760**, 020040 (2016).
23. Martinez-Torres, C., Arneodo, A., Streppa, L., Argoul, P. & Argoul, F. Passive microrheology of soft materials with atomic force microscopy: A wavelet-based spectral analysis. *Appl. Phys. Lett.* **108**, 034102 (2016).
24. Roland, T. *et al.* Revisiting the physical processes of vapodeposited thin gold films on chemically modified glass by atomic force and surface plasmon microscopies. *Surf. Sci.* **603**, 3307–3320 (2009).
25. Khalil, A. *et al.* Chromosome territories have a highly nonspherical morphology and nonrandom positioning. *Chromosom. Res.* **15**, 899–916 (2007).

26. Martinez-Torres, C. *et al.* Diffraction phase microscopy: retrieving phase contours on living cells with a wavelet-based space-scale analysis. *J. Biomed. Opt.* **19**, 036007–036007 (2014).

27. Martinez-Torres, C. *et al.* Deciphering the internal complexity of living cells with quantitative phase microscopy: a multiscale approach. *J. Biomed. Opt.* **20**, 096005–096005 (2015).

28. Mallat, S. & Hwang, W. L. Singularity detection and processing with wavelets. *IEEE Transactions on Inf. Theory* **38**, 617–643 (1992).

Complementary figures

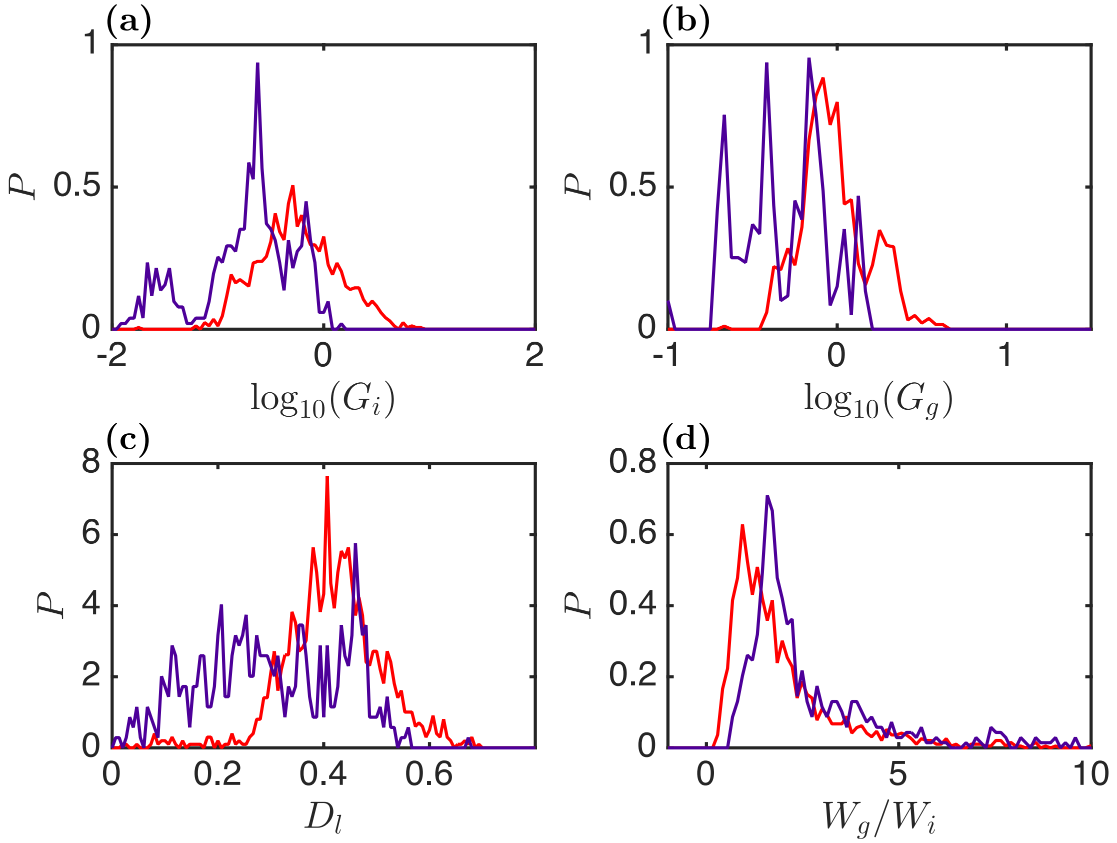

Figure S7. Comparing the distributions of the global mechanical parameters of normal myoblasts (red, 54 cells), myoblasts after blebbistatin treatment (purple, 23 cells). (a) Initial elastic modulus G_i (kPa). (b) Global elastic modulus G_g (kPa). (c) Dissipation loss D_l (Eq. (6)) (see the Section Methods). (d) Ratio of interpolated elastic works W_g and W_i (Eq. (7)).

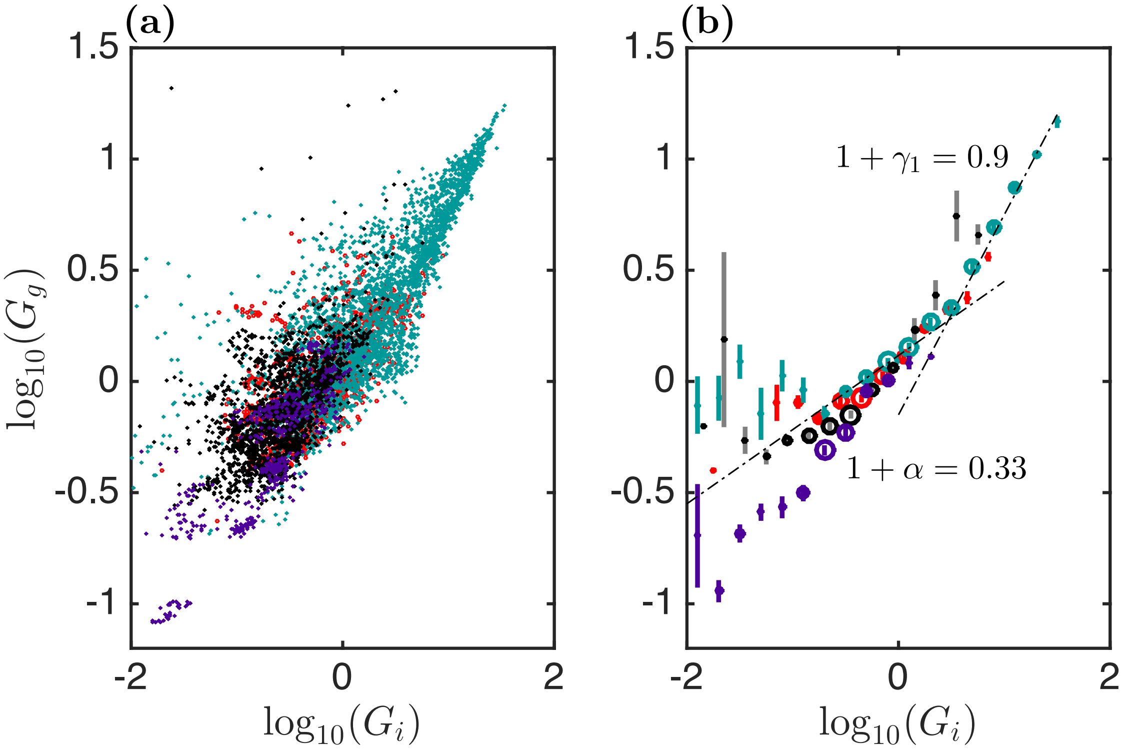

Figure S8. Cross-correlation of the shear moduli G_i and G_g (kPa). These two quantities were estimated from the sets of myoblasts (red), myotubes (blue), ATP depleted (grey) and blebbistatin treated (purple) myoblasts reported in Figs 4 and S7. (a) Scatter plot of $\log_{10}(G_g)$ vs $\log_{10}(G_i)$. (b) Box plots of $\log_{10}(G_g)$ vs $\log_{10}(G_i)$. The vertical lines give the error of the mean of each quantity. The circle diameters are proportional to the percent of FICs with a given G_i.

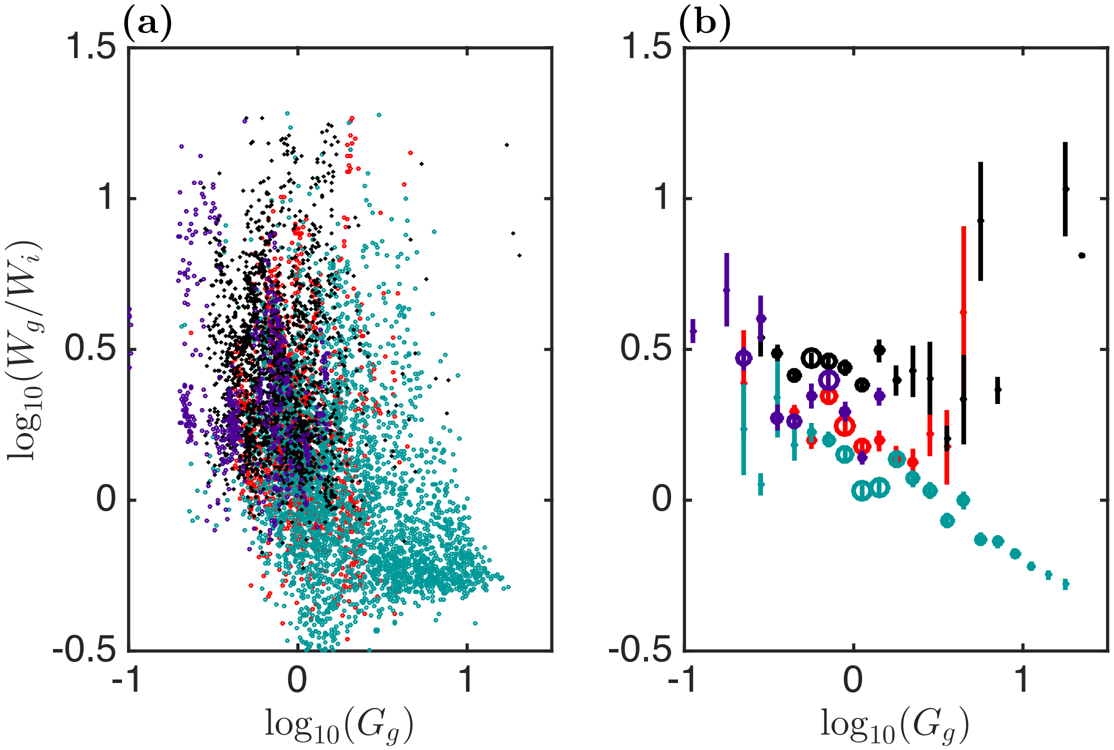

Figure S9. Cross-correlation of W_g/W_i and G_g (kPa). These two quantities were estimated from the sets of myoblasts (red), myotubes (blue), ATP depleted (grey) and blebbistatin treated (purple) myoblasts reported in Figs 4 and S7. (a) Scatter plots of $\log_{10}(W_g/W_i)$ vs $\log_{10}(G_g)$. (b) Box plots of $\log_{10}(W_g/W_i)$ vs $\log_{10}(G_g)$. The vertical lines give the error of the mean of each quantity. The circle diameters are proportional to the percent of FICs with a given G_g.
